# A comprehensive phylogenomic framework for brachiopod evolution among lophotrochozoans

**DOI:** 10.1126/sciadv.aeh5363

**Published:** 2026-10-16

**Authors:** Mingzhu Yang, Aodhán Butler, Natalia López Carranza, Jakob Vinther, Rowan Whittle, Daniela Henkel, Hana Jurikova, Gert Wörheide, Mattia Giacomelli, Philip C. J. Donoghue, Davide Pisani, Sandra J. Carlson, Erik A. Sperling

**Affiliations:** ^1^ Bristol Palaeobiology Group, School of Biological Sciences, University of Bristol, Life Sciences Building, Tyndall Avenue, Bristol BS8 1TQ, UK.; ^2^ Department of Earth and Planetary Sciences, Stanford University, 450 Jane Stanford Way, Stanford, CA 94305, USA.; ^3^ Department of Earth and Planetary Sciences, University of California, Davis, One Shields Avenue, Davis, CA 95616-8605, USA.; ^4^ Bristol Palaeobiology Group, School of Earth Sciences, University of Bristol, Life Sciences Building, Tyndall Avenue, Bristol BS8 1TQ, UK.; ^5^ British Antarctic Survey, High Cross, Madingley Road, Cambridge CB3 0ET, UK.; ^6^ Marine Biogeochemistry/ Marine Geosystems, GEOMAR Helmholtz Centre for Ocean Research Kiel, Wischhofstrasse 1-3, 24148 Kiel, Germany.; ^7^ School of Earth and Environmental Sciences, University of St Andrews, St Andrews KY16 9TS, UK.; ^8^ Department of Earth and Environmental Sciences, Palaeontology and Geobiology, Ludwig-Maximilians-Universität, 80333 München, Germany.; ^9^ GeoBio-Center, Ludwig-Maximilians-Universität, 80333 Munich, Germany.; ^10^ Department of Genetics, Microbiology and Statistics, and Biodiversity Research Institute, Faculty of Biology, University of Barcelona, 08028 Barcelona, Spain.

## Abstract

Brachiopods appeared early in the Cambrian radiation and represent an important clade for understanding the origin of animal body plans and the evolution of biomineralized structures. A robust phylogenetic framework of the living representatives is needed to answer persistent questions about the evolution of brachiopod mineralogy, shell morphology, and identification of the brachiopod sister group among Lophotrochozoa. To the best of our knowledge, we present the first phylogenomic analysis of Brachiopoda and related lophotrochozoan lineages using a comprehensive dataset encompassing all five extant brachiopod orders and using a custom-developed data-refining strategy, compositionally heterogeneous substitution models, and a novel implementation of the parametric-bootstrap likelihood ratio test. We recover monophyly of Lophophorata, with strong support for Brachiopoda as sister to Bryozoa plus Phoronida. Within Brachiopoda, we recover the clade of inarticulated phosphatic Linguliformea sister to the inarticulated calcitic Craniiformea, with both sister to the articulated calcitic Rhynchonellata, with miniaturized Thecideida unexpectedly nested within Terebratulida. Our phylogeny implies that at least one evolutionary transition in primary mineralogy occurred in Brachiopoda, a phenomenon not recognized in any other metazoan group.

## INTRODUCTION

Brachiopoda is a phylum of bivalved, lophophore-bearing animals that are among the earliest extant bilaterians to appear in the fossil record (*1*). The earliest brachiopods are known from the Terreneuvian [early Cambrian (*2*, *3*), 538.8 to ∼521 million years ago (Ma)], and they were important components of the Cambrian (538.8 to 486.9 Ma) and Palaeozoic (538.8 to 251.9 Ma) “evolutionary faunas” (*4*). Brachiopods have one of the highest fidelity fossil records in terms of both completeness and stratigraphic congruence of any major clade (*5*), with more than 12,000 extinct species and ∼410 extant species (according to both BrachNet and WoRMS) representing all major lineages (*6*). While the fossil record provides a robust chronicle of evolution across deep time, incomplete sampling of extant brachiopods has hampered more extensive phylogenomic analysis of this clade.

Brachiopods are unusual among animals in producing distinct primary biomineral types (*7*): The inarticulated Linguliformea mineralize shells of calcium phosphate [apatite (*8*)], while the inarticulated Craniiformea and the articulated Rhynchonelliformea mineralize shells of calcium carbonate [calcite (*9*) or aragonite (*10*)]. Some Linguliformea also mineralize siliceous tablets in their “first formed shells” (*11*, *12*). Major unresolved questions in brachiopod evolution relate to the origin and evolutionary history of the mineralized shell (Fig. 1): What are the key transitions in shell morphology, articulation, and mineralogy, and how robust are the data and analyses that have been hypothesized to explain these transitions?

**Fig. 1.**
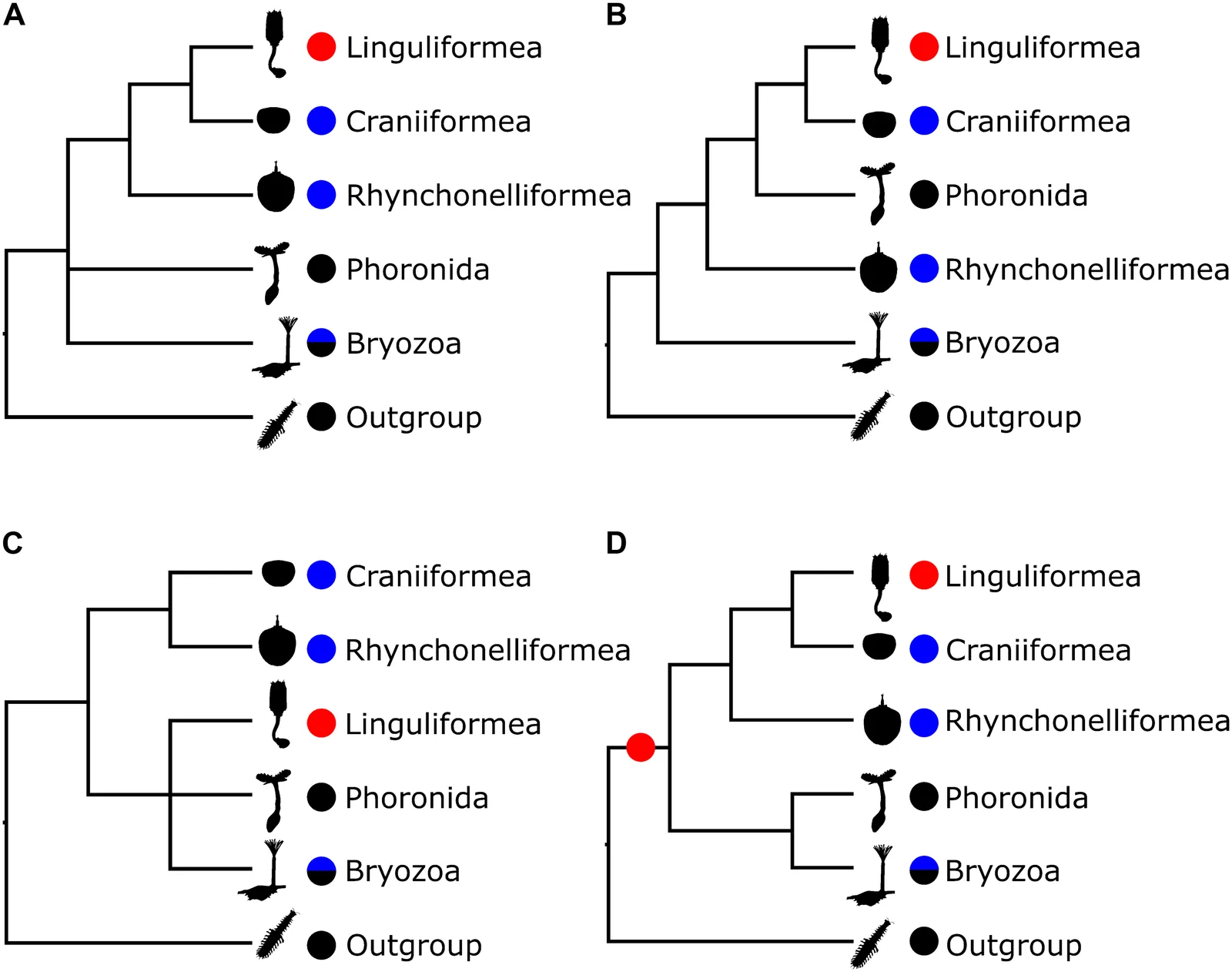
Alternative hypotheses of brachiopod and lophophorate relationships. (**A**) Brachiopod monophyly—Inarticulata/Articulata hypothesis, morphological studies from (*6*, *8*, *14*–*20*) and molecular studies from (*26*–*33*); each clade is monophyletic. (**B**) Brachiopod paraphyly (*23*–*25*, *51*, *77*); phoronids nest within Brachiopoda. (**C**) “Calciata” hypothesis (*21*, *22*); the extant calcareous brachiopods are sisters based on mineralogy. (**D**) “Tommotiid” hypothesis (*18*, *43*, *44*, *48*, *78*, *79*, *103*, *104*); topology after (*48*) and with an ancestral phosphatic scleritome for Lophophorata. Note that multiple phylogenetic hypotheses for extant groups have been aligned with the tommotiid hypothesis. Blue dots represent calcitic mineralogy. Red dots represent phosphatic mineralogy. Black dots represent nonmineralized lineages. Outgroup here refers to other lophotrochozoans. Silhouettes are from https://phylopic.org/. Specific attributions: Riccardo Percudani is the author of Linguliformea (*Lingula anatina*), Guillaume Dera is the author of Rhynchonelliformea (*Anakinetica cumingi*, Davidson 1852); Craniiformea is modified from *Cincinnetina* Jin 2011 created by Matthew Clapham; Martin R. Smith is the author of Phoronida (*Phoronopsis* Gilchrist 1907); Mali’o Kodis is the author of Bryozoa (Ectoprocta); MC Hannon is the author of Annelida. All the abovementioned silhouettes have been released under license CC0 1.0 Universal Public Domain Dedication.

To answer such questions, fossils of early brachiopods and their relatives must be considered within the context of a robust phylogeny of extant groups, such that we can detail the sequence of acquisition and correctly determine polarity/homology of crown-clade characters (*13*). Morphological cladistic analyses have consistently found brachiopods to be monophyletic (*6*, *8*, *14*–*20*), although studies have differed on the pattern of internal relationships, specifically the monophyly of Craniiformea plus Linguliformea (formerly Inarticulata) with respect to the articulated brachiopods (Rhynchonelliformea) (Fig. 1). An alternative topology places the two calcitic brachiopod groups, Craniiformea and Rhynchonelliformea, as sister groups [“Calciata” (*21*, *22*)]. In contrast, analyses of 18*S* and 28*S* ribosomal DNA found Phoronida—a phylum of unmineralized, solitary or colonial lophophorates—nested within Brachiopoda, as sister group to the inarticulates (*23*–*25*), but others found brachiopods to be monophyletic (*26*–*28*). Sperling *et al.* (*29*) noted that these two main alternative topologies are consistent with the same unrooted tree and hypothesized that brachiopod paraphyly in ribosomal trees may be a long-branch attraction artifact caused by relatively rapidly evolving sequences in Rhynchonelliformea. Consistent with this, analysis of a set of seven nuclear housekeeping genes found weak support for brachiopod monophyly in the complete dataset, while the exclusion of fast-evolving sites gave much stronger support for monophyly (*29*). More recent phylogenomic studies have focused on deep Lophotrochozoa/Spiralia relationships and have found brachiopods to be monophyletic (*30*–*33*), but poor taxon sampling within Brachiopoda (e.g., only two to four species) hinders the ability to make this statement with confidence or answer questions regarding evolutionary patterns within the group.

Equally problematic for understanding early brachiopod evolution is confusion about the broader relationships within Lophotrochozoa/Spiralia (*33*–*36*) and the lack of consensus on the brachiopod sister group. In the following, we will use Lophotrochozoa as a synonym for Spiralia to indicate non-ecdysozoan protostomes (*37*). Brachiopods have traditionally been considered as the sister group to Phoronida [“Brachiozoa” (*34*)]. Brachiozoa has then generally been depicted either as a polytomy with other lophotrochozoan phyla (*33*) or as a sister group to Nemertea [“Kryptrochozoa” (*35*)]. The other phylum with a horseshoe-shaped lophophore situated around the mouth, the colonial Bryozoa (Ectoprocta), were traditionally allied with the entoprocts (lacking a true lophophore) and cycliophorans (“Polyzoa”) and were thought to be more distantly related to brachiopods and phoronids. This result has received some molecular support (*31*, *38*), but most other molecular studies, including recent analyses considering homology of ancestral chromosomes and their patterns of fusion and fission, have generally supported a monophyletic Lophophorata consisting of bryozoans, brachiopods, and phoronids (*30*, *32*, *39*, *40*), but see (*36*) for a sobering view of the limits of chromosome fusion applications in phylogenetics. Studies finding monophyletic Lophophorata usually support bryozoans as the sister group of phoronids (*30*, *32*, *39*, *40*), with this clade then as a sister group to Brachiopoda (rather than “Brachiozoa”). Critically, however, multiple studies have demonstrated that systematic errors—driven by heterogeneous evolutionary rates between taxa and compositional heterogeneity across both taxa and sites—strongly affect inferences of lophotrochozoan phylogeny (*31*, *41*). Combined with limited taxon sampling, the sister group of brachiopods remains an open question.

The numerous proposed topologies for the relationships among major extant brachiopod groups, along with uncertainty over the brachiopod sister group, have led to different evolutionary scenarios (not mutually exclusive) for the origin and mineralogical transitions of the shell. These scenarios include transitions from one biomineral type to another within Brachiopoda (*17*, *42*–*48*) (Fig. 1), independent origination of mineralization in brachiopods and bryozoans from nonmineralized ancestors (*49*), convergent evolution of bivalved shells in sister clades within Brachiopoda having different biomineral types (*21*, *50*), and the complete loss of the brachiopod shell in the phoronid stem lineage from mineralized ancestors (*51*, *52*) (Fig. 1).

Phylogenomic strategies have been instrumental in addressing myriad long-standing problems in metazoan phylogeny (*53*, *54*). They offer a promising approach to test conflicting phylogenetic hypotheses of extant brachiopods and phoronids and, ultimately, lead to a better understanding of Cambrian patterns of biomineralization. Brachiopods are one of the few phylum-level groups without a well-sampled phylogenomic framework. This gap reflects practical constraints: Most localities worldwide host only one or two species of brachiopods and often at significant ocean depths, which makes their collection difficult [e.g., (*55*)]. Thus, in contrast to phyla like arthropods, mollusks, or annelids, it is not possible to sample a wide diversity from only a few, shallower sampling localities. We have conducted a global sampling and transcriptomic sequencing campaign that, combined with previously published data, establish the first taxonomically well-sampled transcriptomic dataset for this paleontologically significant phylum and its presumed close relatives.

In reconstructing the phylogeny of Brachiopoda, we took advantage of the Benchmarking Universal Single-Copy Orthologs (BUSCO) software (*56*) and developed a data-refining strategy (Fig. 2) to minimize the potential presence of paralogs and contaminants in BUSCO families. For phylogenomic tree inference, we implemented a diversity of homogeneous and mixture models including CAT-Posterior Mean Site Frequencies (CAT-PMSF) (*57*), which allows approximating Bayesian nonparametric CAT-based models (*58*) in maximum likelihood. We used the Akaike Information Criterion [AIC; see (*59*)] and a new implementation of a parametric bootstrap–based likelihood–ratio (i.e., Goldman) test (*60*) to identify the model that best fit our data, and used topology tests (*61*) to further confirm the fit of the phylogenetic trees inferred in our analyses to the data. These results enable us to test previously proposed hypotheses of brachiopod relationships and establish a robust new framework for studying deep brachiopod evolution.

**Fig. 2.**
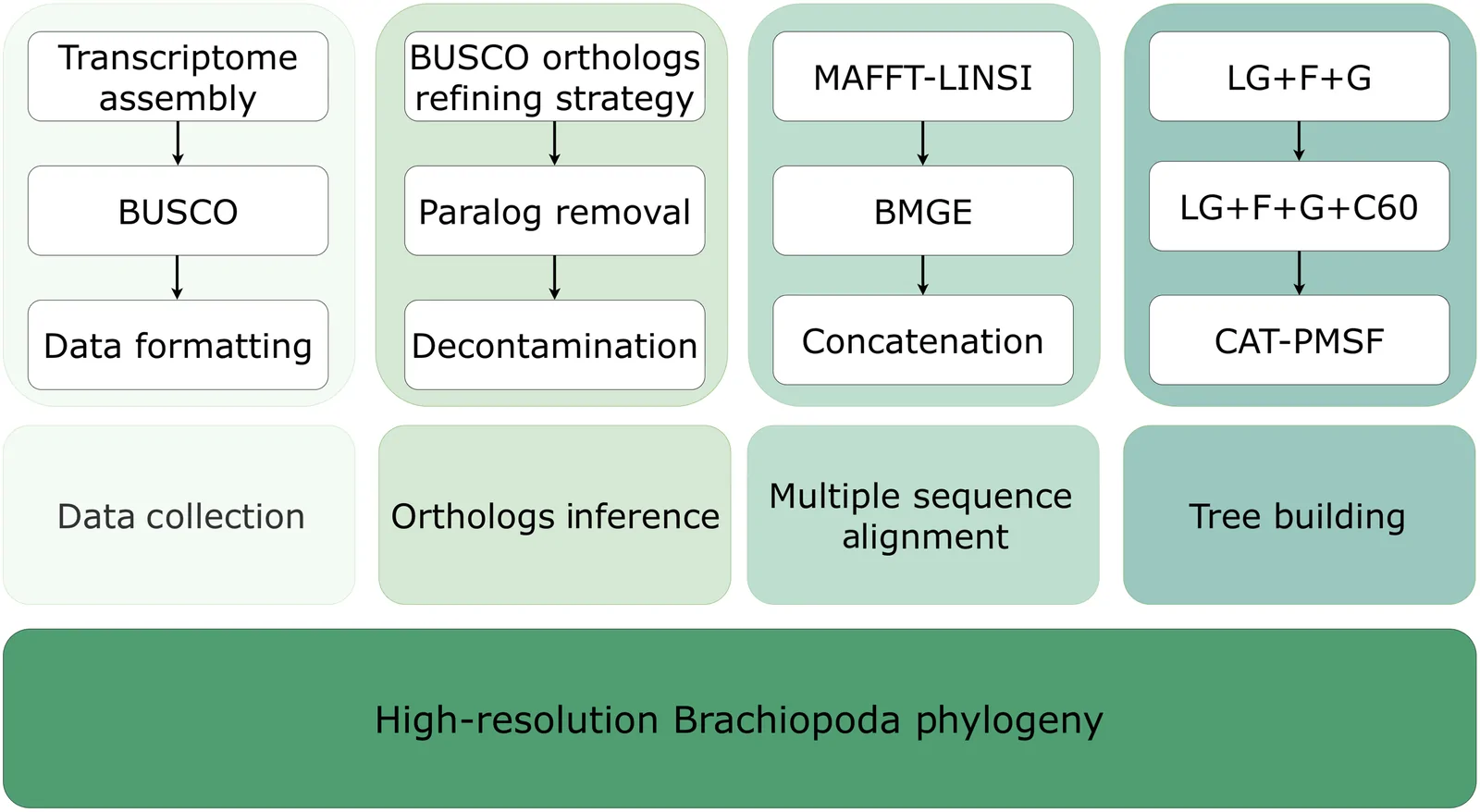
Phylogenomic protocol. Flow chart illustrating the main steps in our analysis.

## RESULTS

### Assembly of a phylogenomic dataset for Brachiopoda

We started from a set of 63 transcriptomes and proteomes, including 18 newly assembled brachiopod transcriptomes covering all five extant orders. From this set of taxa, we assembled a dataset of 672 single-copy genes, which is the number of gene families in the BUSCO-metazoa_odb12 dataset. We used a serial data-cleaning strategy to refine BUSCO gene families (including the removal of families including out-paralogs, thinning of in-paralogs, and removal of hidden-paralogs, possible contaminants, and low-taxon-coverage gene families; see Fig. 2 and Materials and Methods for details), which led to the retention of a total of 301 gene families across 63 taxa. Three taxa were removed because their branch lengths were very long (these were the flatworm *Macrostomum lignano* and the gastrotrichs *Diuronotus aspetos* and *Mesodasys laticaudatus*), causing excessively high levels of phylogenetic instability. The gene families were concatenated into a final super alignment of 93,280 amino acid positions for 60 species.

### Model selection

For our analyses, we considered both across-site compositionally homogeneous and heterogeneous models. Following (*62*), we first performed model-fit tests using AIC in IQ-TREE 2. The results indicate that across-site compositionally heterogeneous mixture models with a fixed number of site frequency categories, specifically the models in the C10 to C60 series (*63*), fit the data better than homogeneous models such as LG+F+G. Among the models tested, LG+F+G+C60 provided the best fit. However, depending on the degree of heterogeneity in the data, nonparametric infinite-mixture models such as CAT-Poisson and CAT-GTR [see (*63*) for an introduction] can outperform LG+F+G+C60, providing a significantly better fit and greater accuracy (*54*, *63*–*65*). These models, however, are not available in IQ-TREE.

CAT-based models are implemented in a Bayesian framework (*58*), in which the model and tree topology are coestimated. For large datasets, however, achieving convergence in Bayesian CAT-based analyses can be challenging. The PMSF approach (*66*) was applied to the case of CAT-based models, leading to the development of CAT-PMSF approach (*57*) to address this problem. Under CAT-PMSF, a CAT model is first inferred in a Bayesian framework on a fixed topology. The PMSF procedure is then used to capture differences in evolutionary patterns among sites by estimating a separate amino acid profile for each site. These site-frequency profiles are saved to an output file, which is subsequently imported into IQ-TREE, where a maximum-likelihood tree search is performed. Separating model parametrization from tree search substantially reduces convergence problems and allows CAT-based models to be applied to larger datasets. However, the CAT-PMSF model implemented in IQ-TREE is an approximation obtained through an averaging process of the CAT models sampled during the Markov chain Monte Carlo (MCMC) in a Bayesian CAT-based analysis, and testing the relative fit of the inferred CAT-PMSF model against other models implemented in IQ-TREE, such as LG+F+G+C60, is key because we cannot assume that CAT-PMSF would fit the data best (*65*). This is problematic because, as the PMSF approach summarizes multiple CAT models (each corresponding to a point along the MCMC chain), into a set of site-specific profiles, the number of parameters of the resulting CAT-PMSF model is not well defined and cannot be precisely enumerated, precluding the direct application of information criteria such as the AIC (*57*, *65*, *66*).

We previously proposed to use parametric bootstrap–based absolute fit tests to compare how well different models capture specific properties of the data, such as across-site compositional heterogeneity (*65*), to compare CAT-PMSF with other models. While informative, this approach is inherently one-dimensional: It evaluates how well the models describe one property of the data at a time. Although a model’s ability to capture a particular property is expected to relate to its overall fit, there is no guarantee that this relationship will be consistent across different properties. Thus, a model that provides the best overall fit may be outperformed by another model with respect to a specific feature of the data, while performing better with respect to other features. Accordingly, Ren *et al.* (*67*) extended the recommendations of Giacomelli *et al.* (*65*) by suggesting that multiple model-adequacy tests should be used to evaluate different features of the data. However, when alternative models differ in their performance across different properties, it becomes difficult, if not impossible, to discriminate between them objectively without reference to a relative fit measure such as the AIC; see (*68*) for an introduction to relative and absolute model-fit tests and their different applications. A more effective, albeit substantially more computationally and memory intensive, approach is the application of a parametric-bootstrap likelihood ratio test (*66*). This approach allows the relative fit of CAT-PMSF to be compared with that of other models, analogous to the use of AIC, without restricting the comparison to specific properties of the data. This approach avoids the problem that the number of parameters in PMSF-based models cannot be readily enumerated (see Materials and Methods for details). Although this approach was proposed by Wang *et al.* (*66*) when the PMSF procedure was originally introduced, it has not been widely implemented because of its high computational cost. With continued advances in high-performance computing, however, parametric-bootstrap likelihood ratio tests should now be feasible for most phylogenomic datasets. We therefore recommend their use (following the steps presented in Materials and Methods and our tutorial—https://github.com/BristolPhylogenomics) over property-specific parametric-bootstrap tests (*65*), which we consider superseded, to compare the fit of CAT-PMSF to that of other models.

Parametric-bootstrap likelihood ratio tests (Fig. 3) demonstrates that, among the three models tested, CAT-PMSF provides the best fit to our dataset (*P* < 0.01). The tests also show that LG+F+G+C60 fits the data better than LG+F+G (Fig. 3A; *P* < 0.01), consistent with the AIC results obtained in IQ-TREE and providing an independent validation of the parametric-bootstrap approach. Comparison of the results for LG+F+G versus LG+F+G+C60 with those obtained for CAT-PMSF versus the two other models (Fig. 3A versus Fig. 3, B and C) provides insight into the magnitude of the differences in model fit. Figure 3A shows that the difference in fit between LG+F+G and LG+F+G+C60 is relatively small: The difference in log likelihood between the two models (Δ*lnL*) observed for the real data (Δ*lnL-real*) is comparable to the distribution obtained for the simulated datasets (Δ*lnL*-*sim*). The Δ*lnL-sim* distribution therefore falls comfortably within the same scale as the observed difference in the real data. In contrast, when CAT-PMSF is compared against LG+F+G or LG+F+G+C60, the difference in fit is substantially larger. The Δ*lnL*-*sim* and Δ*lnL*-*real* values cannot be displayed on a common scale, and the distribution of Δ*lnL*-*sim* is therefore shown as an inset in Fig. 3 (B and C). This indicates that the improvement in fit provided by CAT-PMSF over both LG+F+G+C60 and LG+F+G is not only statistically significant but also substantially greater in magnitude than the improvement observed when LG+F+G+C60 is used instead of LG+F+G. Please see our tutorial (https://github.com/BristolPhylogenomics) for an explanation of all the steps necessary to complete the test performed on a legacy dataset.

**Fig. 3.**
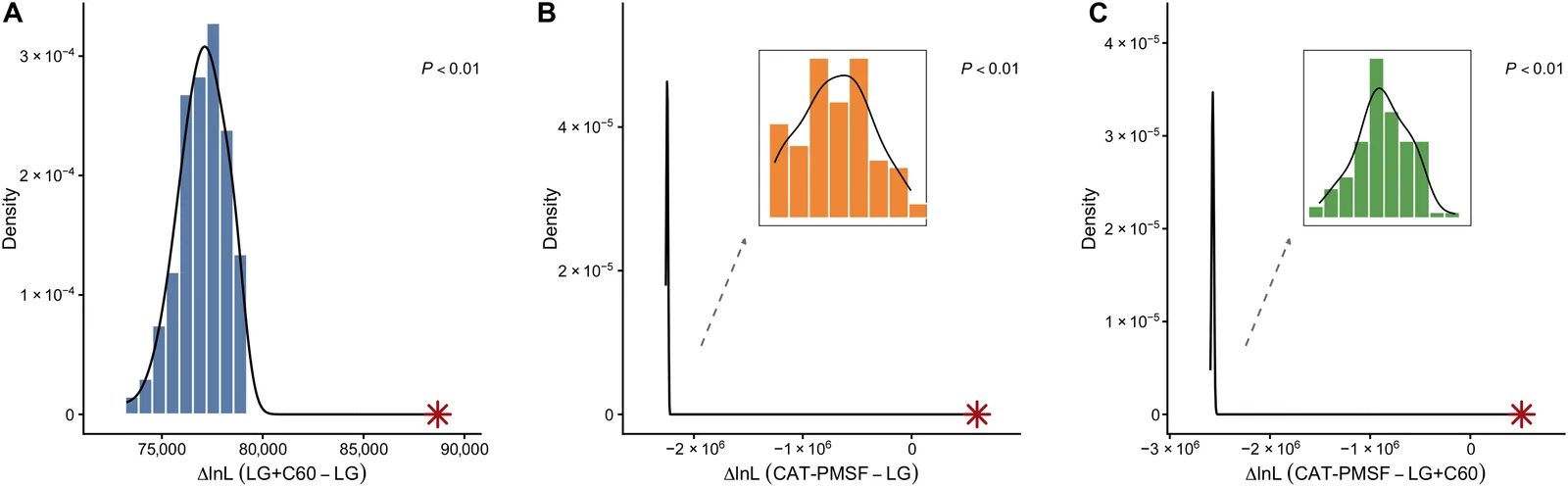
Parametric bootstrap likelihood-ratio tests (Goldman tests) of relative model fit among LG+F+G (LG), LG+F+G+C60 (LG+C60), and CAT-PMSF. (**A**) Under the LG null model, the histogram shows the bootstrap null distribution of Δ*lnL* (LG+C60 – LG). The graph indicates the improvement in fit gained by adding a C60 profile mixture over LG. While the result is significant (*P* < 0.01), the change in fit is of relatively low magnitude as demonstrated by the fact that the value obtained when comparing the two models on real and simulated data could be represented on the same scale. (**B**) Under the same LG null model, the histogram shows the null distribution of Δ*lnL* (CAT-PMSF – LG). The difference in fit is significant (*P* < 0.01), similarly to the case of (A). However, differently from the case of (A), we can see that the difference in magnitude in how LG and CAT-PMSF fit the real and simulated data is so large that the respective Δ*lnL* values cannot be represented on the same scale, with the distribution observed for the simulated data represented in an inset. (**C**) Under the LG+C60 null model, the histogram shows the null distribution of Δ*lnL* (CAT-PMSF – LG+C60). As in (A) and (B), results are significant (*P* < 0.01) and indicate that CAT-PMSF fits significantly better than LG+C60. As in the case of (B), the difference in magnitude between the way in which CAT-PMSF and LG+C60 fit the data is visible as again, Δ*lnL* values cannot be represented on the same scale. Overall, these results show that LG+C60 fits the data better than LG but the difference in fit between the two models is not particularly large. CAT-PMSF fits the data better than both LG+C60 and LG, with the difference in fit being much larger than that between LG and LG+C60.

### Phylogenomic analyses

We performed phylogenomic analyses using all the models mentioned above (LG+F+G, LG+F+G+C60, and CAT-PMSF). It has been shown that results of CAT-PMSF might be mildly affected by the tree topology used to infer the model parameters (*65*). Given ongoing debate surrounding the relationships of some of the lophotrochozoan phyla in our dataset (*30*–*33*), we followed (*65*) and inferred four CAT-PMSF models under four different topologies representing competing hypotheses of lophotrochozoan relationships. These include the tree inferred from our LG+F+G+C60 analysis (Fig. 4B) and the trees from (*30*–*32*); see figs. S4 to S6. Accordingly, we performed four CAT-PMSF analyses and generated a bootstrap consensus tree by merging the ultrafast bootstrap trees from the four CAT-PMSF analyses. We consider the consensus tree to represent our final CAT-PMSF tree (Fig. 5).

**Fig. 4.**
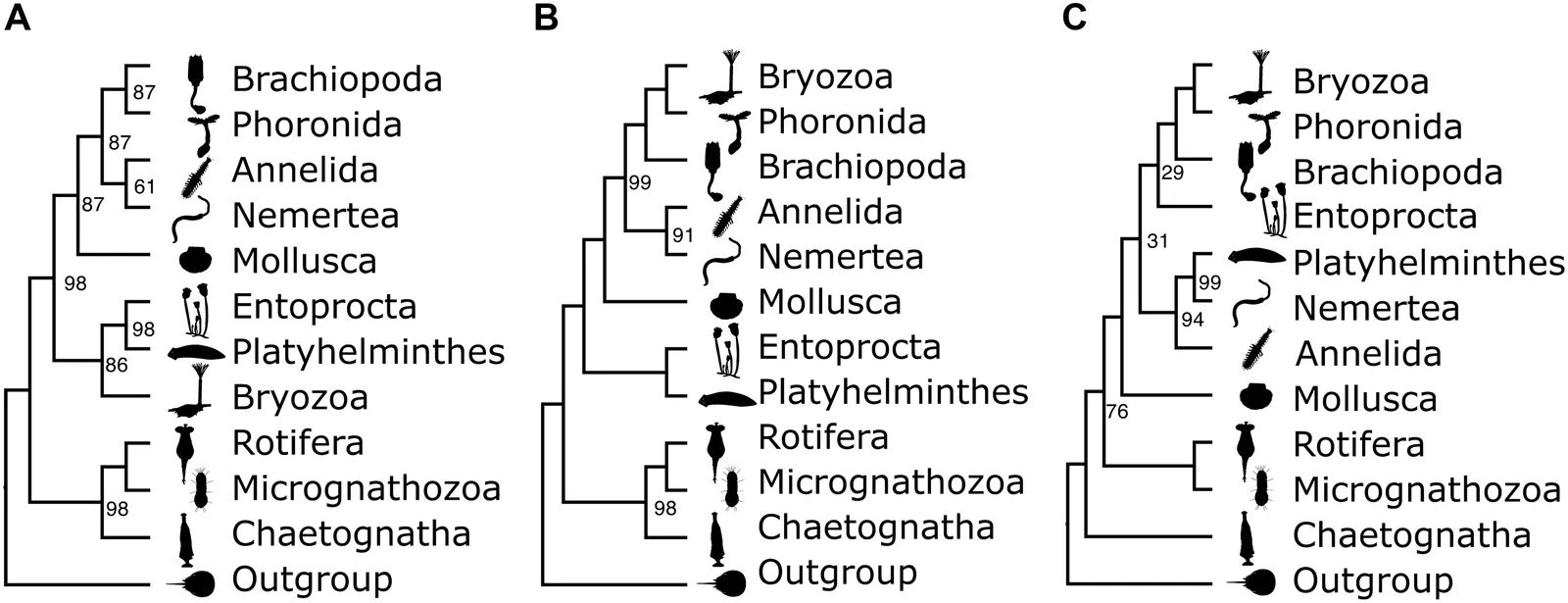
Phylogenetic relationships of Brachiopoda and Lophophorata within Lophotrochozoa under different models. (**A**) Schematic summary of the phylogenetic relationships inferred using LG+F+G. (**B**) Schematic summary of the phylogenetic relationships inferred using LG+F+G+C60. (**C**) Schematic summary of the phylogenetic relationships inferred using CAT-PMSF. (All nodes are 100% supported except for those specifically labeled). Silhouettes are from https://phylopic.org/. Specific attributions: Riccardo Percudani is the author of Brachiopoda (*Lingula anatina*); Martin R. Smith is the author of Phoronida (*Phoronopsis* Gilchrist 1907); Mali’o Kodis is the author of Bryozoa (Ectoprocta); Guillaume Dera is the author of Entoprocta (*Pedicellina choanata* O’Donoghue 1924) and Micrognathozoa (*Limnognathia maerski* Kristensen & Funch 2000); Scott Hartman is the author of Nemertea; Markus Grohme is the author of Platyhelminthes (*Dugesia superioris*, Stocchino & Sluys 2013); MC Hannon is the author of Annelida. Katie Collins is the author of Mollusca (*Pecten maximus*, Linnaeus 1758); Niall McGinty is the author of Chaetognatha (*Eukrohnia*); Andy Wilson is the author of Ecdysozoa (*Limulus polyphemus*). All the above mentioned silhouettes have been released under license CC0 1.0 Universal Public Domain Dedication. T. Michael Keesey is the author of Rotifera (*Rotaria macrura*, Schrank 1803, license link https://creativecommons.org/licenses/by/3.0/). The silhouette of Rotifera was reduced in size.

**Fig. 5.**
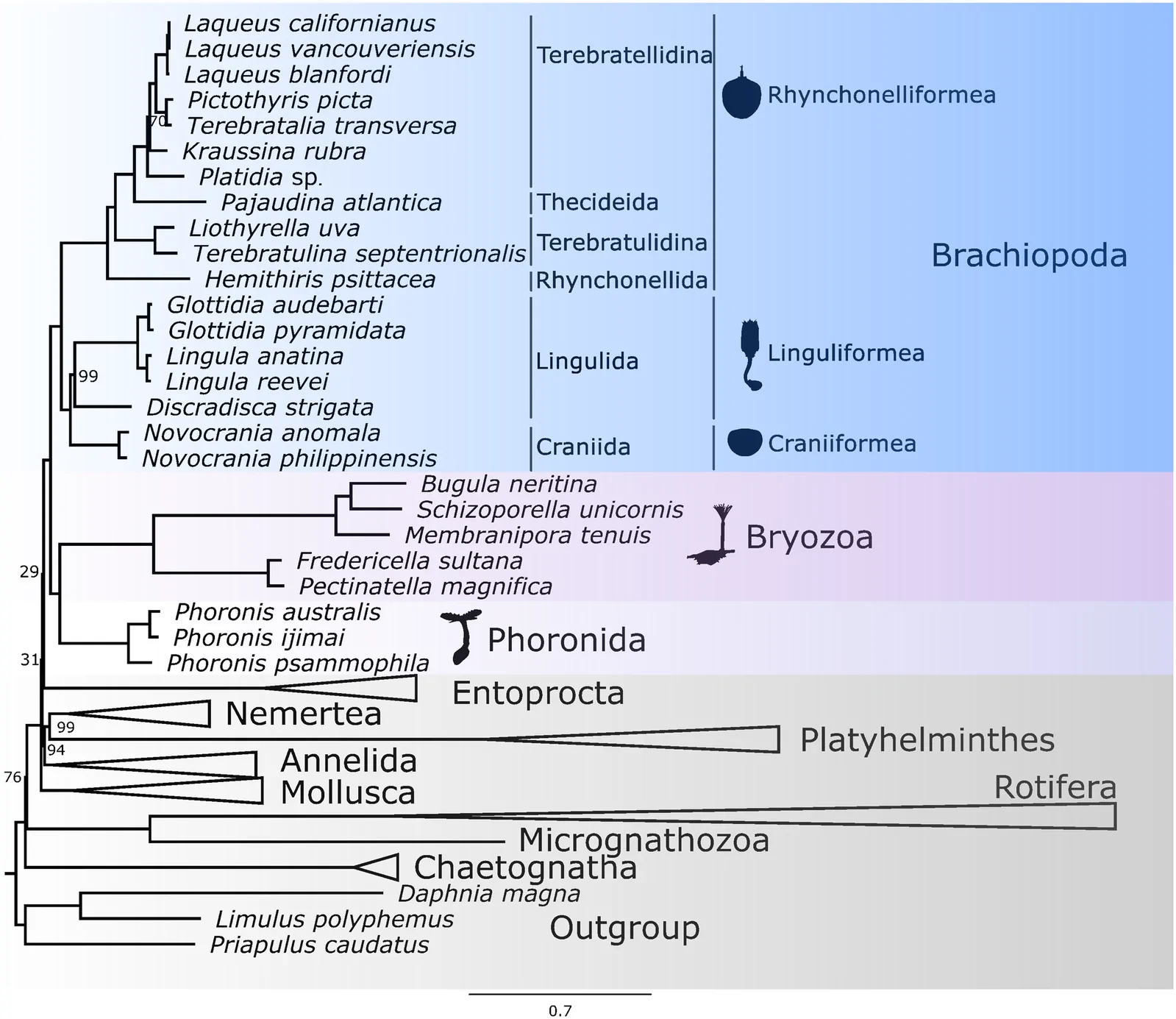
Phylogenetic relationships of Brachiopoda and Lophophorata within Lophotrochozoa under across-site compositional heterogeneous CAT-PMSF model. The CAT-PMSF phylogeny with CUBS values to indicate support for the correspondent node. (All nodes are 100% supported except for those specifically labeled). [Note that specimens previously referred to *Laqueus californianus* are now referred to *L. erythraeus*; see (*105*)]. Silhouettes are from https://phylopic.org/. Specific attributions: Riccardo Percudani is the author of Linguliformea (*Lingula anatina*), Guillaume Dera is the author of Rhynchonelliformea (*Anakinetica cumingi*, Davidson 1852); Craniiformea is modified from *Cincinnetina* Jin 2011 created by Matthew Clapham; Martin R. Smith is the author of Phoronida (*Phoronopsis* Gilchrist 1907); Mali’o Kodis is the author of Bryozoa (Ectoprocta); MC Hannon is the author of Annelida. All the abovementioned silhouettes have been released under license CC0 1.0 Universal Public Domain Dedication.

Branch lengths of the tree in Fig. 5 were reestimated in IQ-TREE using our four CAT-PMSF models. As the branch lengths changed minimally, we present the results with branch lengths reestimated under the CAT-PMSF model inferred using our LG+F+G+C60 tree (which is the CAT-PMSF model used for the parametric bootstrap likelihood ratio tests in Fig. 3). Node support values in Fig. 5 are reported as cumulative ultrafast bootstrap support (CUBS) values, reflecting their derivation from merging the results of our four ultrafast bootstrap analyses. Support values in all the other trees are referred to as standard ultrafast bootstrap support (UBS) values.

All analyses confirm the monophyly of Brachiopoda (Figs. 4 and 5 and figs. S1 to S6), consistent with most previous morphological cladistic analyses and with full support throughout (both UBS and CUBS = 100%). Brachiopods are separated into two monophyletic lineages corresponding to articulated and inarticulated forms, with Rhynchonelliformea sister to Linguliformea plus Craniformea (both UBS and CUBS = 100%; Fig. 5 and figs. S2 to S6).

Within Linguliformea, discinids are confirmed as sister to lingulids, and *Glottidia* and *Lingula* are reciprocally monophyletic. Within Rhynchonelliformea, Rhynchonellida is sister to all other articulated brachiopods. Terebratulidina and Terebratellidina are each monophyletic, and Thecideida is nested within Terebratulida as sister to Terebratellidina (both UBS and CUBS = 100%; Fig. 5 and figs. S2 to S6).

Relationships among lophophorate phyla and other lophotrochozoans emerge as model dependent (Fig. 4 and figs. S1 to S6). The across-site compositionally homogeneous LG+F+G model (Fig. 4A and fig. S1) supports Brachiopoda as sister to Phoronida, and Bryozoa sister to Entoprocta plus Platyhelminthes. In contrast, our LG+F+G+C60 (Fig. 4B and fig. S2) and CAT-PMSF trees (Figs. 4C and 5 and figs. S3 to S6) recover a strongly supported monophyletic Lophophorata (with UBS and CUBS = 100%), where Bryozoa plus Phoronida is the sister of Brachiopoda.

With respect to other lophotrochozoan phyla, our CAT-PMSF tree (Fig. 5) shows Lophophorata in a polytomy with a clade including Entoprocta (CUBS = 29%), Annelida and Nemertea plus Platyhelminthes (CUBS = 31%), and Mollusca (CUBS = 100%). By contrast, LG+F+G+C60 finds Annelida and Nemertea only (UBS = 99%) as sister to Lophophorata, with Mollusca as sister to this combined clade (Lophophorata and Annelida plus Nemertea; UBS = 100%). Deeper in the tree, CAT-PMSF rejects monophyly of the extended Gnathifera clade [see extended Gnathifera terminology in (*32*)], finding Chaetognatha to represent the sister of all the other taxa instead (CUBS = 100%). This contrasts with our LG+F+G and LG+F+G+C60 trees, both of which support Chaetognatha within Gnathifera (UBS = 100%).

We performed approximately unbiased (AU) tests under CAT-PMSF to compare the phylogenetic trees obtained from our different analyses and determine whether any of them are significantly better supported by the data. We found that under all CAT-PMSF models, the topologies inferred using LG+F+G and LG+F+G+C60 are rejected (*P* < 0.01 for all tests, see Table 1). In contrast, the CAT-PMSF tree inferred from the consensus of the bootstrap trees of the four CAT-PMSF analyses is never rejected. This confirms that the CAT-PMSF topology fits the data well under all CAT-PMSF models and significantly better than the topologies from LG+F+G and LG+F+G+C60 models.

**Table 1.** AU tests for topology under different models. LG, the tree topology inferred from the LG+F+G analysis; LG+C60, the tree topology inferred from the LG+F+G+C60 analysis; PMSF-Consensus, the consensus tree inferred merging the ultrafast bootstrap trees from the CAT-PMSF analyses (reported in Fig. 5). CAT-PMSF Model 1: The CAT-PMSF profile inferred under the tree estimated from our LG+F+G+C60 analysis. CAT-PMSF Model 2: The CAT-PMSF profile inferred under the tree topology estimated in the study of (*32*); CAT-PMSF Model 3: The CAT-PMSF profile inferred under the tree estimated in the study of (*31*); CAT-PMSF Model 4: The CAT-PMSF profile inferred under the tree estimated in the study of (*30*).

	Tested topologies		
	LG	LG+C60	PMSF-Consensus
CAT-PMSF Model 1	1.56 × 10^−70^	0.0158	0.984
CAT-PMSF Model 2	5.68 × 10^−6^	4.51 × 10^−10^	1.000
CAT-PMSF Model 3	9.73 × 10^−5^	0.000537	0.999
CAT-PMSF Model 4	8.21 × 10^−7^	0.015	0.985

## DISCUSSION

### Lophotrochozoan relationships and the monophyly of Lophophorata

Analyses of our concatenated dataset invariably found support for the monophyly of Brachiopoda and are consistent in resolving their internal relationships. However, the relationships of Brachiopoda to the other lophotrochozoan taxa and the relationships among lophotrochozoans more broadly depend on the evolutionary models used. All our analyses using across-site compositionally heterogeneous models (Figs. 4 and 5 and figs. S2 to S6) support the monophyly of Lophophorata, with Brachiopoda representing the sister group of Phoronida plus Bryozoa. This result was not recovered when the data were analyzed using the across-site compositionally homogeneous LG+F+G model (Fig. 4A and fig. S1), which instead recovers Brachiopoda as sister to Phoronida, and Bryozoa as the sister to Entoprocta plus Platyhelminthes. Given that AIC and the parametric bootstrap test found that LG+F+G+C60 fit better than LG+F+G and that parametric bootstrap identified CAT-PMSF as best fit, while confirming LG+F+G as worse fit (see above and Fig. 3), our data should be considered to strongly support the monophyly of Lophophorata. This largely confirms the results of (*32*, *39*, *40*), but with substantially expanded sampling of brachiopod species.

Long before the advent of phylogenomic approaches, Valentine (*69*), Strathman (*70*), and Emig (*71*, *72*), among others, argued for the monophyly of Lophophorata on the basis of the possession of a “true” lophophore with coelomic extensions into ciliated tentacles that surround the mouth and exclude the anus. Subsequent analyses using a broader range of morphological and (eventually) molecular traits beyond this one “key” character asserted nonhomology of the lophophore among these three phyla (*73*–*75*). The lophophore in bryozoans was not considered to be strictly homologous with the lophophore in brachiopods and phoronids because of differences in the nature of the coelom and the ciliary band generating water flow through the lophophore (*49*, *73*, *76*). Our phylogenomic results support the earlier view that the lophophore evolved once in the stem lineage of Lophophorata.

Stepping out to consider the sister group to Lophophorata, we found that extremely short internal branches unite Lophophorata (Brachiopoda, Phoronida, and Bryozoa) with Entoprocta, Nemertea plus Platyhelminthes and Annelida, and Mollusca (Fig. 5 and figs. S3 to S6). Consistent with the observation that these branches are very short, support level for the abovementioned nodes is invariably insignificant, even though the clade including all these phyla has CUBS = 100. Also, the other internal branches in our topology are very short, and they are shorter than those in similar trees inferred using CAT-based models within a Bayesian context [e.g., (*32*)]. This suggests that, overall, the lophotrochozoan radiation was rapid and occurred over a short period of time, leaving little signal in genomes to resolve lophotrochozoan relationships. Our results are therefore consistent with those of Serra Silva and Telford (*64*). However, whether this implies that the lophotrochozoan phylogeny might not be resolvable remains, in our opinion, debatable. This is because, for example, there are evident differences between the branches in our tree (Fig. 5) and that of (*32*) that was inferred using a CAT-based model, and these can reflect differences in the sets of genes used by the two studies or differences in the ways in which standard Bayesian CAT-based models and CAT-PMSF model the data: We should note that CAT-PMSF is an approximation of CAT-Poisson.

The short branches in our topology complicate a clear resolution of the sister group relationships of Lophophorata. All our LG-based analyses agree that Annelida plus Nemertea and Mollusca are closely related to Lophophorata [although in the LG tree Lophophorata is to be intended as Brachiopoda plus Phoronida, see different definition of Lophophorata in (*27*, *71*, *72*) (figs. S1 and S2)]. These results are consistent with previous results from (*31*) and the latest molecular analyses of Phoronida (*40*). In contrast, CAT-PMSF (Fig. 5) clusters Platyhelminthes as the sister of Nemertea, and these two as the sister of Annelida, in agreement with (*32*).

Furthermore, our CAT-PMSF analyses suggest that Entoprocta is also a member of the unresolved polytomy that is suggested to include the closest sister to Lophophorata (Fig. 5). This is because all our CAT-PMSF analyses identify a strongly supported, but internally unresolved, clade including Lophophorata, Entoprocta, Annelida plus Nemertea and Platyhelminthes, and Mollusca. While Annelida (plus Nemertea and Platyhelminthes) and Mollusca have previously been suggested to be in the pool of possible lophotrochozoan sister groups to Lophophorata, our results further stress that we have little robust knowledge of this part of animal phylogeny, adding Entoprocta to this list as a possible sister group of Lophophorata. However, as we did not optimize taxon sampling to address relationships deep in the lophotrochozoan phylogeny, we can only conclude that further analyses, with an expanded taxon sampling, will be necessary to improve resolution within Lophotrochozoa.

### Brachiopod monophyly and biomineralization

Considering brachiopod monophyly and the fundamental relationships within Brachiopoda, with wider taxon and gene sampling our results convincingly overturn previous molecular analyses based on ribosomal genes suggesting that brachiopods were paraphyletic with respect to phoronids (*23*–*25*, *51*, *77*). The results of the phylogenomic analyses broadly agree with some previous morphological cladistic analyses (*6*, *8*, *14*–*20*) in placing Craniiformea and Linguliformea as reciprocal sister groups with respect to Rhynchonelliformea. In addition, our results support the homology of valve articulation across brachiopods, with articulation present as a shared feature of Rhynchonelliformea and absent in Craniiformea plus Linguliformea (“Inarticulata”). However, because the polarity of these two states cannot be determined, neither can be considered as a synapomorphy.

Based on analyses of extant taxa alone, our phylogenomic topology indicates that the brachiopod shell was originally calcitic, with an organophosphatic mineralogy evolving from a calcitic mineralogy later in the Linguliformea. A phosphatic ancestry could still be implied under the hypothesized “tommotiid origin” scenario (*43*, *44*, *48*, *78*, *79*), in which several different lower Cambrian phosphatic tommotiids are placed within the stem group of Brachiopoda and other lophophorate phyla (*48*), with calcitic mineralogy evolving convergently in Rhynchonelliformea and Craniiformea (Fig. 1D). Our phylogenomic results support the biomineralization interpretations discussed here, but they do not allow us to reject all other possible alternatives, and a comprehensive evaluation of the polarity of mineralogy among lophotrochozoans awaits testing with fossil data that is beyond the scope of this study. Nonetheless, assuming homology of the brachiopod shell, our phylogenomic results imply that at least one transition in primary skeletal mineralogy of the shell occurred within Brachiopoda, and possibly more than one transition.

In contrast to brachiopods, other animal lineages evolved an initial skeletal mineralogy typically reflecting the most favorable seawater chemistry (*80*, *81*) that was largely retained throughout their evolutionary history. Some mollusks, such as bivalves, have switched between different calcium carbonate polymorphs (calcite versus aragonite). Porifera (sponges) is the phylum with most clearly distinct chemical compositions of their skeletal elements, with different groups producing siliceous (Demospongiae, Hexactinellida, and Homoscleromorpha) versus calcitic (Calcarea) spicules. Other sponge groups, such as the extinct stromatoporoids and extant sclerosponges, build accretionary calcium carbonate skeletons (sometimes with siliceous spicules as well). However, the processes of biosilicification and biocalcification and possibly even biophosphatization in sponges are complex, and available evidence indicate that they evolved independently (*82*). Thus, these appear to represent (as far as known) independent acquisitions of a new biomineralized structure using a different biomineral (as is also the case for the evolution of calcareous otoliths in vertebrates or the de novo evolution of siliceous tablets in discinid brachiopod larval shells). This differs from the brachiopod shell, where at least one of the major brachiopod lineages appears to have changed the fundamental biomineral type used in the construction of a homologous structure; this has not been recognized in other metazoan clades. While polarizing the precise direction of mineralogical change will require a total-evidence analysis integrating Cambrian fossils with our robust phylogenetic tree of extant taxa, the fundamental mineralogical transition identified by our phylogenomic analysis points to a potentially unique Cambrian evolutionary event within brachiopods.

### Phylogenetic relationships within Brachiopoda: Implications for morphology and body size evolution within Terebratulida

Our expanded sampling within the crown group Rhynchonelliformea supports each of the four major subgroups: Rhynchonellida, Terebratulidina, Terebratellidina, and Thecideida (Fig. 5). Rhynchonellida (*Hemithiris*), which is also the first of these groups to appear in the fossil record, have spirolophe lophophores as adults, supported only posteriorly by small, mineralized hook-shaped crura. Spirolophes lacking any mineralized support are present in adult inarticulated brachiopods (*Glottidia*, *Lingula*, *Discradisca*, and *Novocrania*) and appear to represent the ancestral state for brachiopods, from which other lophophore types evolved (*6*). Among these extant brachiopods, rhynchonellides are the sister group to Terebratulida, which is distinguished by a more geometrically complex plectolophe lophophore supported by mineralized structures extended from crura, known as “loops.” So-called short-looped terebratulides (*Liothyrella* and *Terebratulina*) have delta-shaped loops, while long loops (e.g., in *Laqueus*) are more complex in three dimensions. The differences in loop geometry are also supported here as homologs with phylogenomic data.

Within Terebratulida, several interesting results were obtained regarding body size and lophophore support. Among terebratulides with the smallest adult body size (2 to 4 mm in length) that we sampled, *Platidia*, classified as a “long-looped” terebratulide but with an unusual arc-shaped loop, is the sister group to the rest of the terebratellidine clade. *Platidia* first appears in the fossil record post-Cretaceous (100.5 to 66 Ma) and is thought to be derived and paedomorphic (*55*, *83*, *84*) rather than the sister group to other larger-bodied (10 to 50+ mm in length) long-looped terebratulides like *Laqueus* and *Terebratalia*. *Kraussina*, which here is sister to the other more typical terebratellidines, first appears in the Pliocene (5.33 to 2.58 Ma) and is larger than *Platidia* in body size but also has a highly unusual double-pronged lophophore support despite being classified as a long-looped terebratellidine. Kraussinoids, many of which are very small-bodied, are also considered to represent late occurring and derived terebratellidines (*85*).

In contrast to previous analyses of morphology, we found Thecideida (*Pajaudina*) to be nested within Terebratulida, as the sister group to the long-looped terebratellidine terebratulides and not the sister group to all Terebratulida (*23*, *86*) nor sister to all other extant articulated brachiopods (*17*). As appears to be the case for small-bodied *Platidia*, phylogenomic results place thecideides (1 to 3 mm in length) in a basal position relative to larger-bodied terebratellidines, as the sister group to the larger-bodied short-looped terebratulidines. Greatly reduced body size in the very small-bodied thecideide and platidioid brachiopods was necessarily accompanied by major evolutionary changes in morphology. Several other groups of extant small-bodied terebratulides with atypical lophophore supports are thought to be paedomorphic and derived [e.g., Megathyridoidea and Gwynioidea (*85*)]. It is possible that the increasingly small volume of the mantle cavity cannot accommodate the inclusion of a relatively rigid, three-dimensional mineralized lophophore support. Other possibilities are that hydrostatic pressure in the coelom is sufficient to support the tentacles in the small trocholophe or schizolophe lophophore or that mineralized spicules, common in the lophophores of these very small terebratulides, provide sufficient support and flexibility. Investigating the phylogenomic position of a broader sample of these very small-bodied terebratulides will allow us to test whether small body size is phylogenetically ancestral among terebratellidine brachiopods or has evolved progenetically multiple times within the long-looped clade. Heterochrony, specifically progenesis, might have driven the repeated evolution of very small body size in brachiopods and other metazoans, potentially leading to unexpected and possibly misleading morphological phylogenetic results (*7*, *87*).

### Morphological evolution in Lophophorata: Implications for function and ecology

On the basis of the CAT-PMSF tree (Fig. 5), we can conclude that Lophophorata has undergone a succession of evolutionary morphological innovations. First, the lophophore—a ciliated tentacular organ containing coelomic extensions that encloses the mouth and excludes the anus—originated only once within Lophophorata, a clade comprising three monophyletic phyla: brachiopods, bryozoans, and phoronids. Ciliary action on the lophophore tentacles generates and directs water flow and is thus critical to respiration, feeding, and reproduction in these organisms (*47*, *88*, *89*). Second, mineralization in Lophophorata occurs in the bryozoans and brachiopods only, which appears to enable and enhance morphological diversification, particularly in the bivalved brachiopods. Homology and polarity of transformation in mineralization and mineralogy remain somewhat uncertain, although the current phylogenomic topology requires at least one major change in primary mineralogy during brachiopod evolution. Third, only brachiopods, solitary individuals of larger body size, have a mineralized bivalved shell creating a mantle cavity that encloses the lophophore (*8*, *47*). This body plan significantly influences the lophophore function, distinguishing brachiopods from the smaller, often colonial bryozoans and phoronids. In extant brachiopods, the water flow is generally directed in through the sides through the gape of the valve and out the central anterior gape, after being processed for oxygen and food. Fourth, the two brachiopod sister clades are distinguished by the presence or absence of articulation between the valves (*29*). The difference in articulation influences extant brachiopod lifestyle and ecology: Many linguliforms are free-living and burrow in sediment; craniiforms and thecideides are cemented to a hard substrate; rhynchonelliforms and some linguliforms live attached to a hard substrate by a nonmineralized pedicle. Fifth, the derived rhynchonellates (*8*, *90*, *91*) sampled here have a mineralized support for the lophophore in the form of crura, loops, or brachial ridges (*6*, *17*), which appear to have enabled the diversification of lophophore geometries, from the basal spirolophe (Rhynchonellida, also Linguliformea and Craniiformea) to plectolophe (Terebratulida) and ptycholophe (Thecideida), thereby influencing lophophore function. Last, coordinated morphological transformations occurred several times within the terebratulides, the most diverse group of extant brachiopods, concurrent with greatly reduced body size, likely through the paedomorphic process of progenesis (*83*). The phylogenomic data unexpectedly supports thecideides (*Pajaudina*) nesting within the terebratulide brachiopods (*23*), consistent with this observation. Although traditionally classified as an order separate from the order Terebratulida, Thecideida may represent a previously unrecognized example of selection for reduced body size among post-Paleozoic terebratulides, accompanied by numerous morphological modifications that obscure relationships inferred from morphology alone, underscoring the crucial value of phylogenomic analysis.

### Summary

By combining transcriptome-scale phylogenomic data with rigorous orthology inference and broad taxon sampling, we establish, to our knowledge, the first comprehensive and well-supported phylogenetic framework for Brachiopoda among other lophotrochozoans. Our study confirms homology of the lophophore and monophyly of Lophophorata and reinforces the Brachiopoda sister-group relationship with Bryozoa plus Phoronida. Furthermore, we resolve internal relationships within Brachiopoda, confirming its monophyly by uniting the articulated and inarticulated clades, each monophyletic themselves. Significantly, our study clarifies mineralogical evolution among extant Brachiopoda, in depicting a likely transition in biomineralogy of a homologous structure, which has not been recognized previously among metazoans.

From their evolutionary origin in the Cambrian, through their dominance on the Paleozoic seafloor, to their continued, albeit less diverse, presence in the modern ocean that enables molecular, developmental, ecological, and physiological studies, brachiopods represent one of the best clades for integrating paleontological, morphological, and molecular studies of evolutionary history. As with many other clades, phylogenomic analysis has established a robust backbone topology for brachiopods, providing a foundation that will underpin future studies while also revealing numerous intriguing questions regarding their evolutionary history.

## MATERIALS AND METHODS

### Specimen collection

Live brachiopod and bryozoan specimens (marine in all cases) were collected from the United States, Canada, Antarctica (South Orkney Islands), Japan, Panama and Spain, by a combination of hand collection in the intertidal zone, collection by scuba diving below the low-tide level, through dredging, or by purchase from marine supply companies (table S5). All necessary permits for the sample collection in this study were obtained. Organisms were carefully removed from their environment with scalpel blades and placed straight into chilled (5°C) RNAlater or ethanol if not immediately processed.

### Transcriptome sequencing and assembly

#### *RNA extraction*, *library generation, and sequencing*

Dissection of the mantle tissue and viscera was carefully performed on externally cleaned brachiopod valves under a stereo microscope with care taken to avoid inclusion of gut contents and other external environmental contamination. In most cases, RNA was extracted with a Qiagen RNA kit using silica spin columns, but in challenging samples, a phenol-chloroform RNA extraction protocol with TRIzol was used to maximize yield and quality in samples where polysaccharide and mucus compounds made the usual spin column method difficult to perform. An additional deoxyribonuclease enzyme processing step was performed to remove contamination from remaining genomic DNA. Extracted RNA was assayed on a NanoDrop spectrograph analyzer to check the initial quality and quantity of extracted RNA at each stage of the extraction procedure. For library preparation, extracted RNA was shipped to the Huntsman Institute on dry ice overnight and then quality checked on an Agilent Technologies 2200 TapeStation. Most cDNA libraries were then prepared with an Illumina TruSeq cDNA kit. Individual libraries were normalized to 10 nM, and equal volumes were pooled in preparation for Illumina sequence analysis. Sequencing libraries (25 pM) were chemically denatured and applied to an Illumina HiSeq v4 paired end flow cell using an Illumina cBot. Hybridized molecules were clonally amplified and annealed to sequencing primers with reagents from an Illumina HiSeq PE Cluster Kit v4-cBot (PE-401-4001). Following transfer of the flow cell to an Illumina HiSeq 2500 instrument (HCS v2.2.38 and RTA v1.18.61), a 125-cycle paired-end sequence run was performed using HiSeq SBS Kit v4 sequencing reagents (FC-401-4003). Several libraries were also prepared using the Illumina TruSeq Stranded mRNA Library Prep kit (20020595) and TruSeq RNA UD Indexes (20022371). These libraries were then normalized to 1.30 nM in preparation for Illumina NovaSeq sequence analysis. On each lane on a NovaSeq S4 flow cell (2 to 2.5B read pairs per cell), a 30-μl volume of a 1.15 nM pool of libraries was added. Following transfer of the flow cell to an Illumina NovaSeq 6000 instrument, a paired-end sequence run [2 × 150 base pairs (bp)] was performed to a depth of ∼50 to 60 million reads per sample. We note that for some species, we sequenced multiple individuals and only used those with best overall quality for our analyses.

#### Transcriptome assembly and completeness evaluation

Transcriptomic data from 18 brachiopod individuals (from 13 species) and 2 bryozoan individuals (2 species) generated in this study were assembled using the following approach. Raw reads were processed with Trimmomatic v0.36 (*92*) to remove TruSeq sequencing adapters and trim low-quality bases (Phred score < 3) from the leading and trailing ends of reads, retaining reads with a minimum length of 36 bp. Trimmed reads were subsequently assessed using FastQC (*93*). De novo assembly of the reads was performed using Trinity v2.4.0 (*94*) on the LMU supercomputing cluster and the Bridges computing cluster. Assembly quality was evaluated using standard transcriptome metrics, including N50, N90, and L50. Following assembly, protein sequences were predicted using a custom Python script (prottrans.py by W. Francis). Short protein sequences (<100 to 150 amino acids) were discarded. Completeness was assessed using the core eukaryotic conserved gene dataset (*95*) with CEGMA and later with BUSCO as an additional quality control step.

Three Brachiopoda transcriptomes (SRR2005824 *Terebratalia transversa*, SRR2131392 *Kraussina rubra*, and SRR1611557 *Laqueus californianus*) were assembled using the following approach. Raw sequencing reads were quality controlled with fastp v0.20.0 (*96*), and transcripts were de novo assembled with Trinity v2.13.2. Coding sequences were extracted, predicted, and translated using TransDecoder v5.5.0. Single-copy genes were predicted using BUSCO v5.8.3, and a completeness scoring was calculated for each proteome. 2 brachiopod proteomes (*Glottidia pyramidata* and *Novocrania anomala*) were obtained directly from Fernández *et al.* (*97*). The rest of the proteomes were downloaded from National Center for Biotechnology Information (NCBI) RefSeq database; see detailed sources in table S1.

### Phylogenomic dataset construction

We started from a set of 63 transcriptomes and proteomes including 18 brachiopod species, representing all five orders—see table S1 for a list of all the taxa in the dataset and accession numbers.

To assemble our phylogenomic datasets, we used BUSCO on our proteomes to extract the BUSCO single-copy orthologous genes, assembling an initial dataset of 672 single-copy orthologous genes from the BUSCO-metazoa_odb12. BUSCO uses a simple HMM-threshold-based criterion to assign individual sequences to single-copy orthologous families, which performs well on evaluating completeness but does not generate ready-made phylogenomic datasets. It is relatively easy to show that BUSCO families might include sequences with very heterogeneous branch lengths (e.g., fig. S7), suggesting the potential presence of paralogs. To address this problem, we developed an in-house approach that uses a set of reference genome–derived proteomes (see table S2) to subsample and sanitize BUSCO families. The output of this approach is a BUSCO-based dataset that includes a subset of validated BUSCO families, reducing the potential inclusion of paralogs in the phylogenomic dataset.

#### Core dataset assembly: Assembling a refined BUSCO-based phylogenomic dataset

We selected 12 taxa spanning the whole of Metazoa, confident that they had high-quality genomes (see table S2 for details), to build an initial reference database. For this set of 12 taxa, we performed both BUSCO v5.8.3 and OrthoFinder I (hereafter OrthoFinder) analyses (*98*). The 672 BUSCO families were then compared against the results from OrthoFinder. Our reasoning is that while both BUSCO and OrthoFinder can be expected to make errors when inferring single-gene orthologous families, families that are found to be composed exclusively of single-gene orthologs by both approaches are more likely to be valid single-gene orthologous families.

However, BUSCO and OrthoFinder results cannot be compared in a straightforward manner because the two software programs use idiosyncratic definitions. For example, BUSCO defines a single-copy orthologous family as every gene family that did not undergo duplications, irrespective of whether it underwent deletions, whereas OrthoFinder single-copy orthologous families are those that did not undergo duplications or deletions. First, we identified all families that are identified as single-copy by both BUSCO and OrthoFinder; these were included in our phylogenomic dataset. After that, we identified all BUSCO families with one-to-many relationships relative to OrthoFinder families. These are families that are defined differently by the two protocols. As there is no certainty as to whether the BUSCO or the OrthoFinder delimitation is correct, these families were deemed inadequate for phylogenetic reconstruction and excluded. We next identified all BUSCO families with a one-to-one relationship against families that OrthoFinder defines to be multicopy. For each of these families a series of checks were made to evaluate whether they could be deemed phylogenetically useful or not. First, we checked which of these BUSCO families were classified by OrthoFinder as multicopy gene families that only underwent gene losses. All such families were retained as valid single-gene families, as there is no direct evidence that they underwent duplications. Last, all BUSCO families with a one-to-one correspondence to OrthoFinder multicopy gene families where duplications can be unquestionably identified were separated into two groups. The first included all BUSCO families that corresponded to a OrthoFinder multicopy gene family where only in-paralogs of terminal taxa could be identified. The second included all BUSCO families where at least one out-paralog (with reference to terminal taxa) could be identified. All families in the second group were automatically excluded. BUSCO families matching OrthoFinder multicopy gene families where only the presence of in-paralogs of terminal taxa were identified, were further screened. For each OrthoFinder family including in-paralogs for one or more terminal taxa, we screened the corresponding BUSCO family and tested whether the BUSCO sequence was present in the OrthoFinder family, i.e., we tested whether the BUSCO sequence was one of the in-paralogs. When that was the case, the BUSCO family was deemed adequate to be included in our phylogenetic dataset. This is because all in-paralogs have the same orthology relationship to the other sequences in the dataset, and they are all equally valid orthologue representative for the considered species. Families where the BUSCO sequence did not match one of the corresponding in-paralogs were excluded. This screening process removed a total of 268 BUSCO families that were deemed as having the potential to include paralogs, with our final dataset of reciprocally validated families including 404 putative single-gene families distributed across a subset of 12 high-quality reference metazoan genomes.

#### Accretion phase: Dataset expansion and paralog removal from the final 63-taxon dataset

A final dataset of 404 BUSCO families was then created by adding the 63 target taxa that were not already included in our initial 12-species dataset. This was done by adding the sequence identified by the BUSCO pipeline for each of the 404 retained families in our set of 63 transcriptomes and proteomes of interest. We note that because the 12 reference genomes had to span all the Metazoa (as we used the Metazoa BUSCO dataset), our final dataset included 75 taxa. However, taxa that were used to define our families but were not of phylogenetic interest for this study were removed (before building multiple sequence alignments).

We are confident that the sequences for our 404 families should be orthologs for our set of 12 reference genomes. However, the accretion phase might have added hidden paralogs. This is because the BUSCO threshold-based approach might still cause the inclusion of paralogs across the newly added taxa. Accordingly, a second sanitation step was completed to minimize the inclusion of potential paralogs. For this step we used a BLAST-based approach as a heuristic shortcut to establish the orthology of the newly integrated sequences. We started from a large dataset of 217 publicly available metazoan proteomes (see table S6 for a list of medians for each family) that we maintain in-house, and that includes all the 63 taxa that we wanted to include in our final dataset. This dataset was used to create an initial ranking (within each family) to understand the quality of the relevant sequences that were included. To achieve this goal, the HMM-bitscore of each sequence in each family was collated and a family HMM-bitscore ranking was generated. After that, we calculated the median bitscore for each family and identified for which one of the 12 reference taxa the retained sequence had a bitscore above the median. This step was done to confirm that, for each family, the sequence retained from the 12-reference genome had a low probability to represent a paralog. If these sequences were paralogs they would be expected to have a low bitscore because of their greater evolutionary distance from the orthologous members of the considered family. All sequences for all 12 reference genomes with a bitscore above median within their family were included in a new blast database and a series of BLAST analyses were performed against this database. More precisely, every sequence added in the accretion phase was BLASTed against this new reference sequence database. Newly added sequences were validated as members of the considered family only if they hit every sequence retained (for that family), across the subset of 12 taxa retained in the new reference sequence (i.e., above median) database. Sequences added in the accretion phase that failed to hit one or more of the sequences from the core set of genomes (with above median bitscore) were rejected. This step resulted in the exclusion of ∼6% of sequences across our 404 families.

#### Decontamination phase

The approach described above (generation of the initial dataset and the subsequent accretion phase) was designed to remove paralogs but will fail to identify contaminants if these are orthologs of considered genes. We then added a final step in our dataset generation protocol to minimize the potential inclusion of contaminants. For this step, we used a BLAST-based approach. First, we built a new reference dataset (see list of taxa in table S3) including the taxa in our dataset and a diversity of key taxa not represented in our dataset but representing either putative contaminants or taxa belonging in the same lineage as the taxa in our dataset. The protocol used a two-step approach. For the first step, the taxa added to our blast database were as follows: two fungi (*Podila verticillata*, *Spizellomyces punctatus*), two Ichthyosporea (*Amoebidium parasiticum*, *Sphaeroforma arctica*), one Filasterea (*Capsaspora owczarzaki*), two Choanoflagellata (*Salpingoeca dolichothecata*, *Salpingoeca urceolata*), one Porifera (*Oscarella pearsei*), two Ctenophora (*Lampetia pancerina*, *Mnemiopsis leidyi*), two Cnidaria (*Acropora millepora*, *Actinia tenebrosa*), two Placozoa (*Hoilungia hongkongensis*, *Trichoplax adhaerens*), six Bilateria (*Daphnia magna*, *Hermodice carunculata*, *Limulus polyphemus*, *Ciona intestinalis*, *Lineus longissimus*, *Sipunculus nudus*). BLAST analyses were run for each of the sequences in our dataset against this new reference dataset. For each tested sequence, we extracted taxonomic information for each target sequence that was hit with an e-value <1 × 10^−20^. We compared the taxonomic information of the top nonself hit and made sure it was a member of Bilateria. If it was not, the sequence was excluded as a potential contaminant. In the second step we used a reference database that included two Brachiopoda (*Pictothyris picta*, *Pajaudina atlantica*), two Bryozoa (*Schizoporella unicornis*, *Pectinatella magnifica*), two Entoprocta (*Barentsia gracilis*, *Pedicellina cernua*), two Phoronida (*Phoronis australis*, *Phoronis psammophila*). This second round focused on the Lophophorata more explicitly plus the Entoprocta that in some older classifications were considered to be related to Bryozoa. In this second analysis all sequences for Brachiopoda that did not have a best (nonself) hit against Brachiopoda were excluded as potential contaminants. Similarly, for Bryozoa, Phoronida, and Entoprocta sequences that did not have a match (for the best nonself hit) against, respectively, Bryozoa, Phoronida and Entoprocta were excluded. BLAST-based contaminant removal excluded ∼8% of the sequences across our whole dataset.

#### Removal of low-taxon-coverage families

A final filtering step was performed where families that, after the paralog-removal and decontamination steps, covered less than 50% of the taxa in our full dataset were excluded. This step led to the final exclusion of 103 BUSCO families. This was done to reduce potential negative effects caused by missing data. Overall, after these three steps, we were left with a total of 301 families (see finalized BUSCO family list in table S4).

### Multiple sequence alignment and gene tree inspection

The final set of 301 curated families were aligned using MAFFT (v7.526, L-INS-i) (*99*) with default parameters. Single-gene trees were built using IQ-TREE 2 (v2.4.0) (*62*), under best-fitting models selected by ModelFinder, including site-heterogeneous mixture models with fixed numbers of categories (C10-C60). Analyses of the single-gene families identified three problematic species that are invariably associated with very long branches in our dataset. These were the flatworm *M. lignano* and the gastrotrichs *D. aspetos* and *M. laticaudatus*. These species were excluded from our dataset such that our final version included 60 taxa. The final alignment was curated using BMGE (v1.12) (*100*) with default parameters. All curated alignments were concatenated into a final superalignment of 60 taxa and 93,280 amino acid positions, which is available in a Zenodo repository (https://doi.org/10.5281/zenodo.19209727).

### Phylogenetic analysis and model testing

#### Tree inference

We performed a diversity of analyses on our super alignment. First, we used the across-site compositionally homogeneous LG+F+G model, as implemented in IQ-TREE 2 (*62*). After that, the data were analyzed using across-site compositionally heterogeneous models. We first used the best-fitting across-site compositionally heterogeneous model with fixed number of categories (*58*) available in IQ-TREE 2. Model selection was completed using ModelFinder in IQ-TREE using the AIC. The selected best-fitting model was LG+F+G+C60. Last, we used the CAT-PMSF (*57*, *65*) approach, which allows porting infinite mixture models to maximum likelihood inference. To implement CAT-PMSF, we followed the approach in (*65*) and parameterized CAT-Poisson models in PhyloBayes (*101*) under four different fixed topologies. This was done because the tree topology used to infer the CAT-PMSF model might have a mild biasing effect on CAT-PMSF analyses (*65*). As lophotrochozoan (sensu lato) relationships are still debated, we preferred not to choose a single lophotrochozoan tree to estimate our CAT-PMSF model as this might have biased our results in favor of that phylogeny and against alternative hypotheses of lophotrochozoan relationships. The four trees used to infer our CAT-Poisson models were our LG+F+G+60 tree and three different trees obtained from the literature (*30*–*32*). When inferring our CAT-Poisson models in PhyloBayes, we assessed convergence using *tracecomp*, ensuring that all effective sample size (ESS) were >100 and the relative difference (reldif) was <0.3 for all the parameters. We extracted the site profiles using the *readpb_mpi* -ss command, sampling 100 points of the posterior parameter space. Then, we converted site profiles into a site frequency file using the script *convert_site.py* (*57*). In this way, we generated four site-frequency profiles that were used to perform four different CAT-PMSF analyses in IQ-TREE 2. Support values were estimated using the ultrafast bootstrap for all four CAT-PMSF analyses. Following (*65*), we then generated a composite CAT-PMSF bootstrap tree merging the set of ultrafast bootstrap trees from the four individual CAT-PMSF analyses. The branch lengths of the composite CAT-PMSF tree were reestimated under each one of the CAT-PMSF models in IQ-TREE 2.

#### Topology testing under AU

We used the AU test (*61*) to compare the fit of the composite CAT-PMSF tree against that of the trees inferred in individual CAT-PMSF analyses. This was done to make sure that the composite CAT-PMSF tree was not suboptimal under any of the CAT-PMSF models that we used. In addition, we tested the fit of the composite CAT-PMSF tree against that of the alternative hypotheses of lophotrochozoan relationships used to infer the CAT-PMSF models (*30*–*32*).

#### Parametric-bootstrap likelihood ratio test of relative model fit

The parametric-bootstrap likelihood-ratio framework described by Wang *et al.* (*66*), following the general approach of Goldman (*60*), was used to statistically compare CAT-PMSF against both LG+F+G+C60 and LG+F+G and to compare LG+F+G+C60 against LG+F+G. The second test (LG+F+G+C60 against LG+F+G) is strictly speaking redundant as we already use AIC to compare these models. However, the latter test serves to validate our results as we expect the AIC and the parametric bootstrap test to agree and provides a useful reference for interpreting the relative fit of CAT-PMSF by comparing LG+F+G+C60 against both CAT-PMSF and LG+F+G.

The parametric-bootstrap likelihood ratio test compares two models at a time and evaluates whether the difference in likelihood they achieve on the real data can be ascribed to a chance event. We provide a tutorial, using a test dataset, and including scripts to complete each step in the analysis here: https://github.com/BristolPhylogenomics. Here, we will describe in detail how the test was implemented to compare CAT-PMSF and LG+F+G+C60. After that, we will illustrate in what way the test needs to be changed to compare CAT-PMSF against LG+F+G and LG+F+G+C60 against LG+F+G. However, for commands and scripts used to run the test, please read: https://github.com/BristolPhylogenomics.

First, the model achieving the higher likelihood on the real data is identified. In the case of the comparison between CAT-PMSF and LG+F+G+C60, that was CAT-PMSF. The likelihood is then reestimated (for both models) on the tree preferred by the model that achieved the lower likelihood (in our case, the LG+F+G+C60 tree). This is because, in the test, the lower likelihood model (LG+F+G+C60) and the tree inferred under that model are the null model and the null tree. The difference between the log likelihoods of the compared models, a quantity referred to as Δ*lnL-real* is then calculated as

$$\begin{array}{c} \text{ΔlnL}‐\text{real}=\text{lnL}‐\text{real}\left(\text{CAT}‐\text{PMSF},T_{\text{null}}\right)\\ -\text{lnL}‐\text{real}\left(\text{LG}+F+G+C60,T_{\text{null}}\right) \end{array}$$

A specified number of datasets is then simulated; in our study, we simulated 100 datasets (*n* = 100). The simulated datasets must have the same size as the real dataset and are generated by parametric bootstrap (*59*, *60*) under the *null* model and the *null* tree in AliSim (*102*). Each simulated dataset is then analyzed (this was done in IQ-TREE 2) under the *null* model, fixing the *null* tree. Parameters that in the *null* model are free to vary (the component weights and +G) are reestimated for each simulated dataset, and the likelihood is recorded. After that, each simulated dataset is analyzed (this was done in IQ-TREE 2) under a “frozen” CAT-PMSF model. That is, a CAT-PMSF model using the site-frequency profiles estimated from the real data (no reestimation of the site-frequency profiles is performed in this step). The likelihood achieved by the “frozen” CAT-PMSF model on the simulated datasets is recorded, and for each simulated dataset, the difference between the log likelihoods of the two models, a quantity we refer to as Δ*lnL-sim*,*i* (where *i* is the simulation number), is computed

$$\begin{array}{c} \Delta \mathrm{lnL}‐\mathrm{sim},i=\mathrm{lnL}‐\mathrm{sim},i\left(\text{CAT}‐\text{PMSF},\text{frozen},T_{\text{null}}\right)\\ -\mathrm{lnL}‐\mathrm{sim},i\left(\text{LG}+F+G+C60\;\text{reestimated},T_{\text{null}}\right) \end{array}$$

The proportion of cases where Δ*lnL-sim*,*i* ≥ Δ*lnL-real* is used to estimate the probability, under the null model, that the model achieving higher likelihood on the real data (i.e., CAT-PMSF) will obtain on the simulated data an advantage at least as large as observed in the analysis of the real data. This is done by calculating

p=(r+1)/(N+1)

We then calculate *r* as the proportion of cases where Δ*lnL-sim*,*i* ≥ Δ*lnL-real*. The null model is rejected when *P* < 0.05, indicating that the observed advantage of the higher-likelihood model would be unexpected if the data were a product of the null model.

The rationale behind the test is that if the real data really were just an ordinary product of the null model, then the observed advantage of the higher-likelihood model (CAT-PMSF) could have arisen by chance. This is tested by evaluating the propensity for the same (hence frozen) site-frequency profiles to outperform the *null* model when the latter is the true data-generating model. The test therefore asks how often, with LG+F+G+C60 simulated data, CAT-PMSF achieves an advantage at least as large as that observed in the real data. If that almost never happens the real data’s preference for the higher-likelihood model is unlikely to be a chance event, and we can conclude that the higher-likelihood model (CAT-PMSF) captures features of the data that the *null* model does not capture.

When testing CAT-PMSF against LG+F+G or LG+F+G+C60 against the LG+F+G, the same steps were taken, with the difference that LG+F+G became the *null* model for both tests, and the tree preferred by LG+F+G became the *null* tree, as both CAT-PMSF and LG+F+G+C60 achieved a higher likelihood than LG+F+G for the real data. A tutorial explaining how to calculate this test is available here: https://github.com/BristolPhylogenomics.
