# Supplementary material for: A comprehensive phylogenomic framework for brachiopod evolution among lophotrochozoans
Source: Sci Adv. 2026 Oct 16;12(42):eaeh5363. doi: 10.1126/sciadv.aeh5363 (PMC13674499; doi:10.1126/sciadv.aeh5363)
Supplement: Supplementary file 1 [file sciadv.aeh5363_sm.pdf]

Science Advances
AAAS

Supplementary Materials for

A comprehensive phylogenomic framework for brachiopod evolution among lophotrochozoans

Mingzhu Yang *et al.*

Corresponding author: Davide Pisani, davide.pisani@bristol.ac.uk; Sandra J. Carlson, sjcarlson@ucdavis.edu; Erik A. Sperling, esper@stanford.edu

Sci. Adv. **12**, eaeh5363 (2026)
DOI: 10.1126/sciadv.aeh5363

The PDF file includes:

Figs. S1 to S9
Legends for tables S1 to S6

Other Supplementary Material for this manuscript includes the following:

Tables S1 to S6

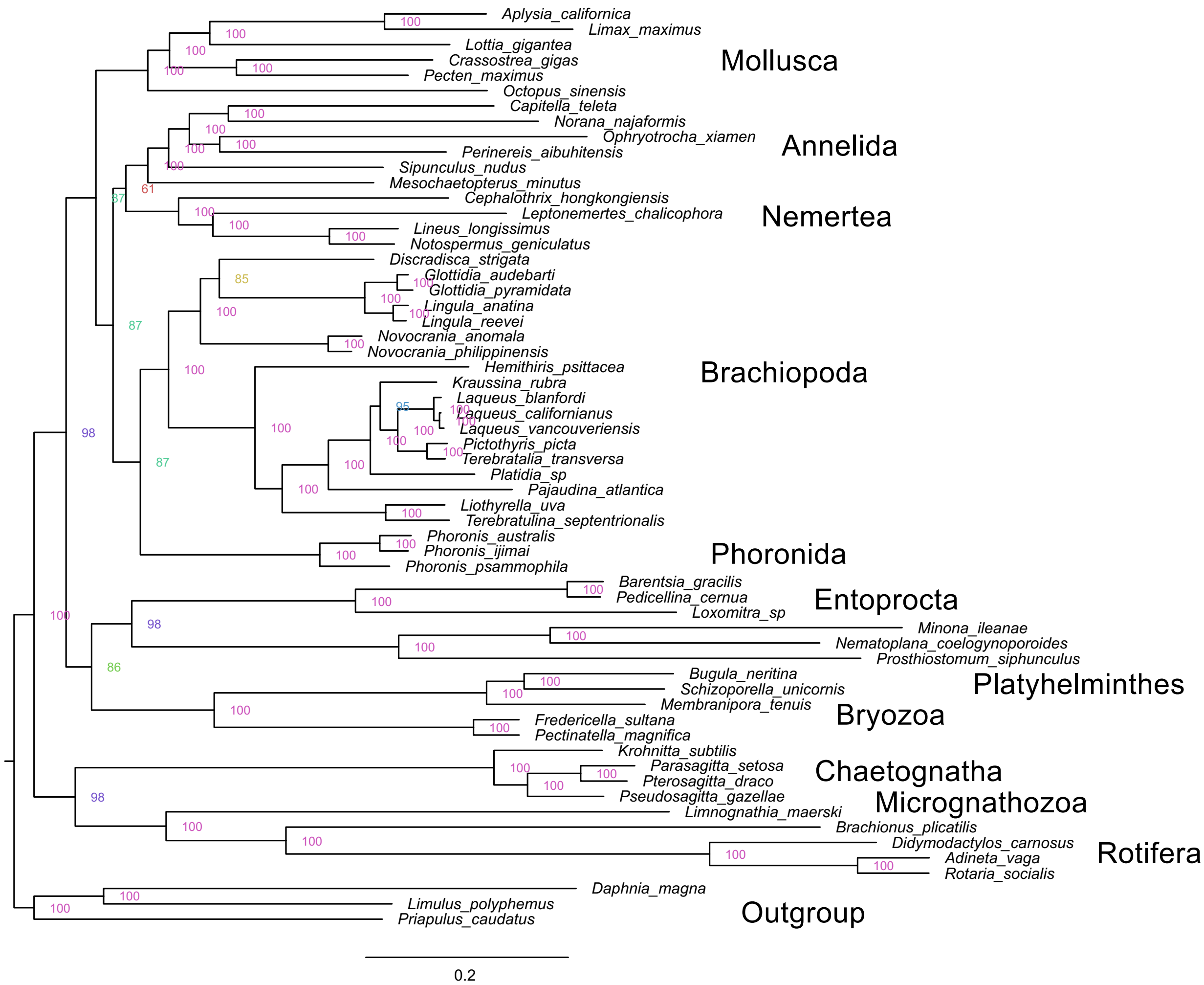

Fig. S1 Phylogenetic topology of Brachiopoda and Lophophorata within Lophotrochozoa under across-site compositional homogeneous LG+F+G model.

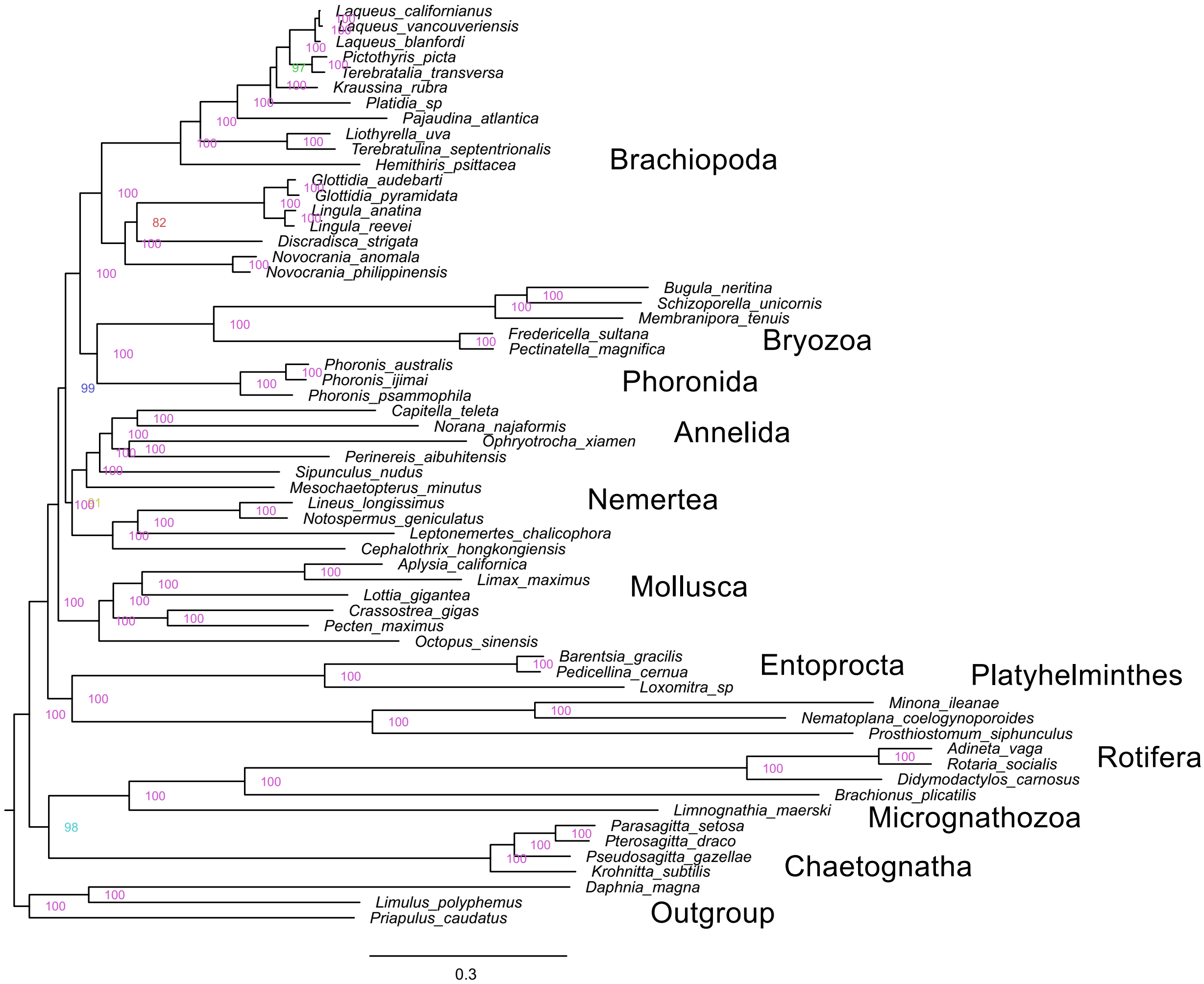

Fig. S2 Phylogenetic topology of Brachiopoda and Lophophorata within Lophotrochozoa under across-site compositional heterogeneous LG+F+G+C60 model.

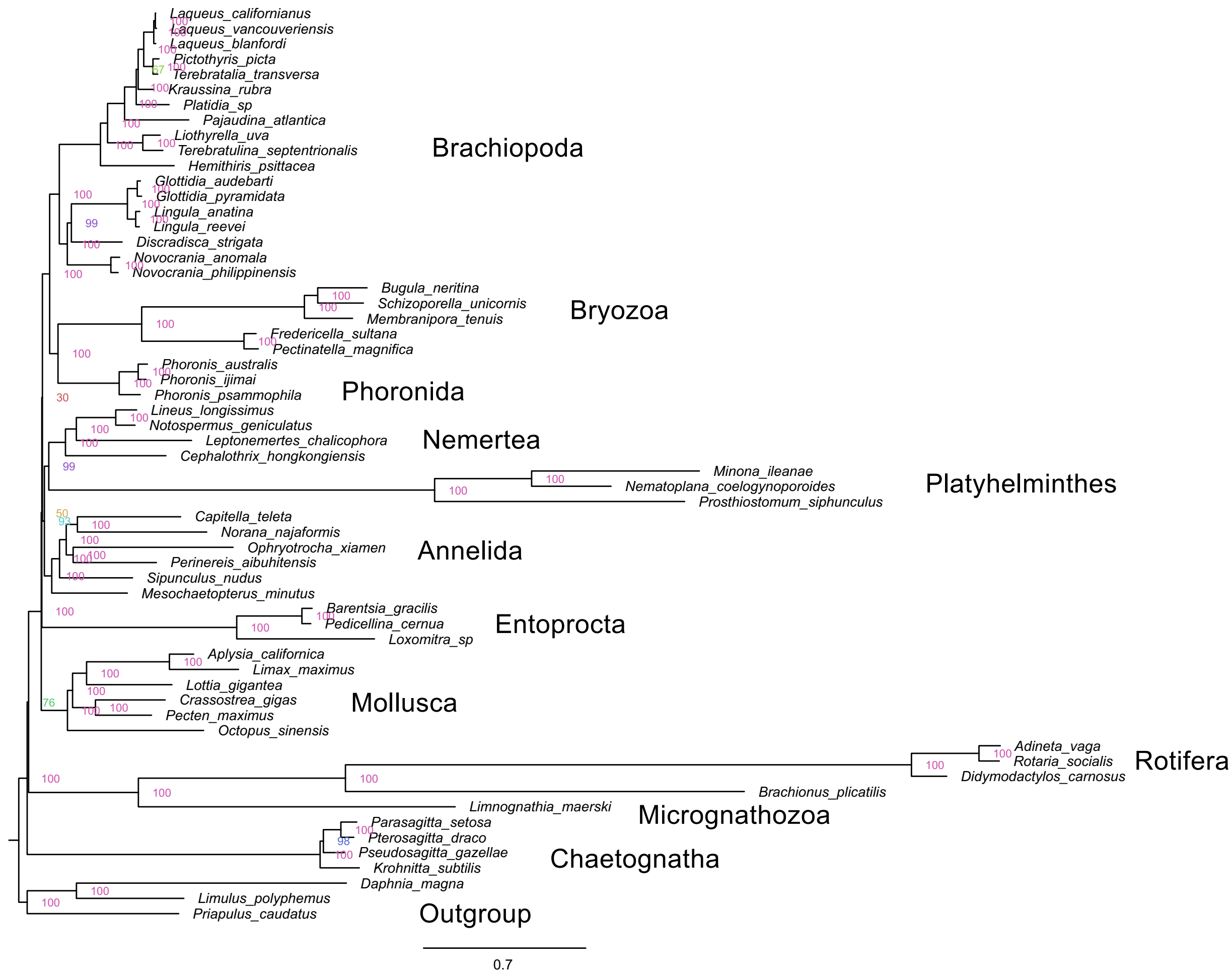

Fig. S3 Phylogenetic topology of Brachiopoda and Lophophorata within Lophotrochozoa under across-site compositional heterogeneous infinite CAT-PMSF model under LG+F+G+C60 topology.

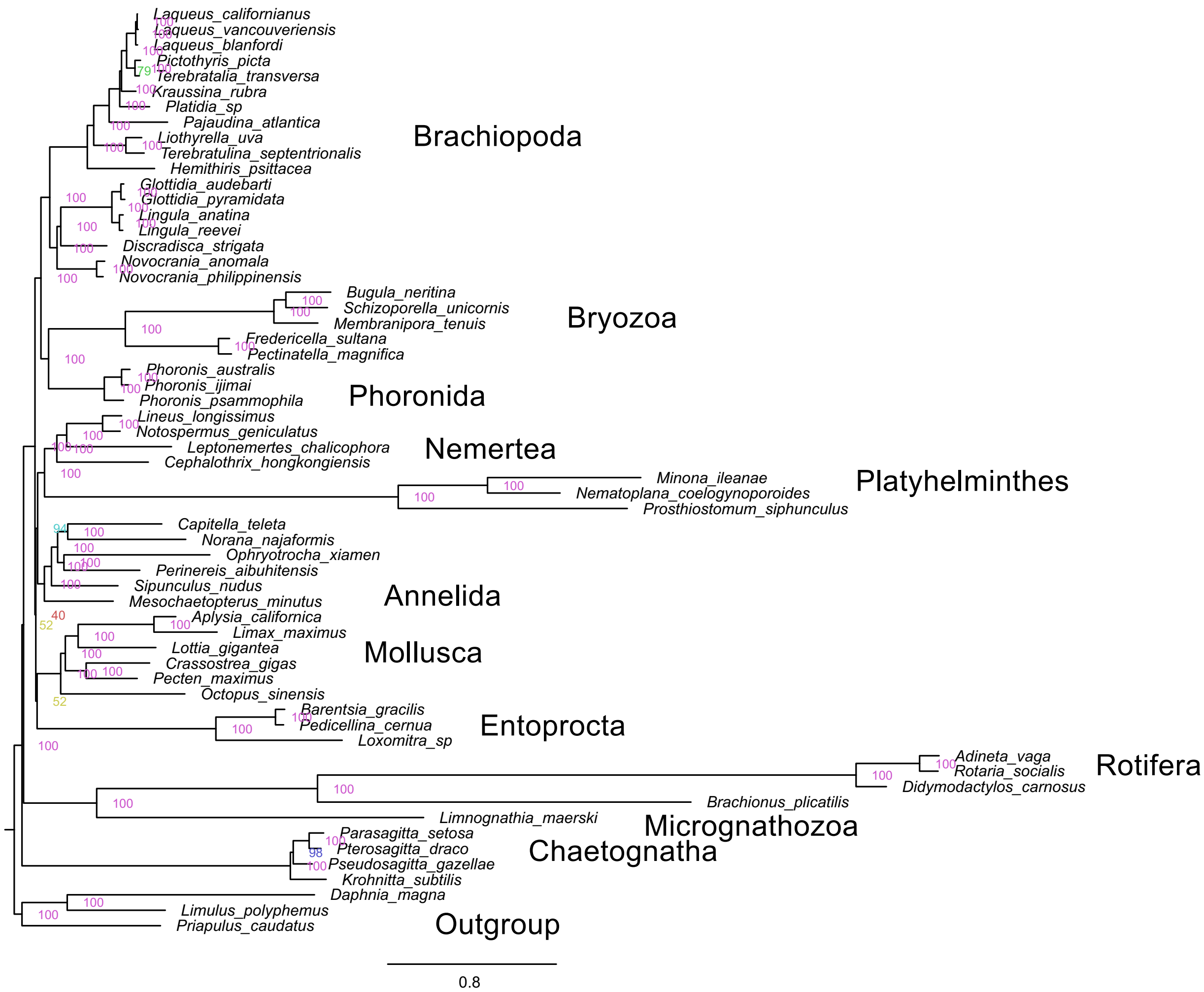

Fig. S4 Phylogenetic topology of Brachiopoda and Lophophorata within Lophotrochozoa under across-site compositional heterogeneous infinite CAT-PMSF model under (*32*) topology.

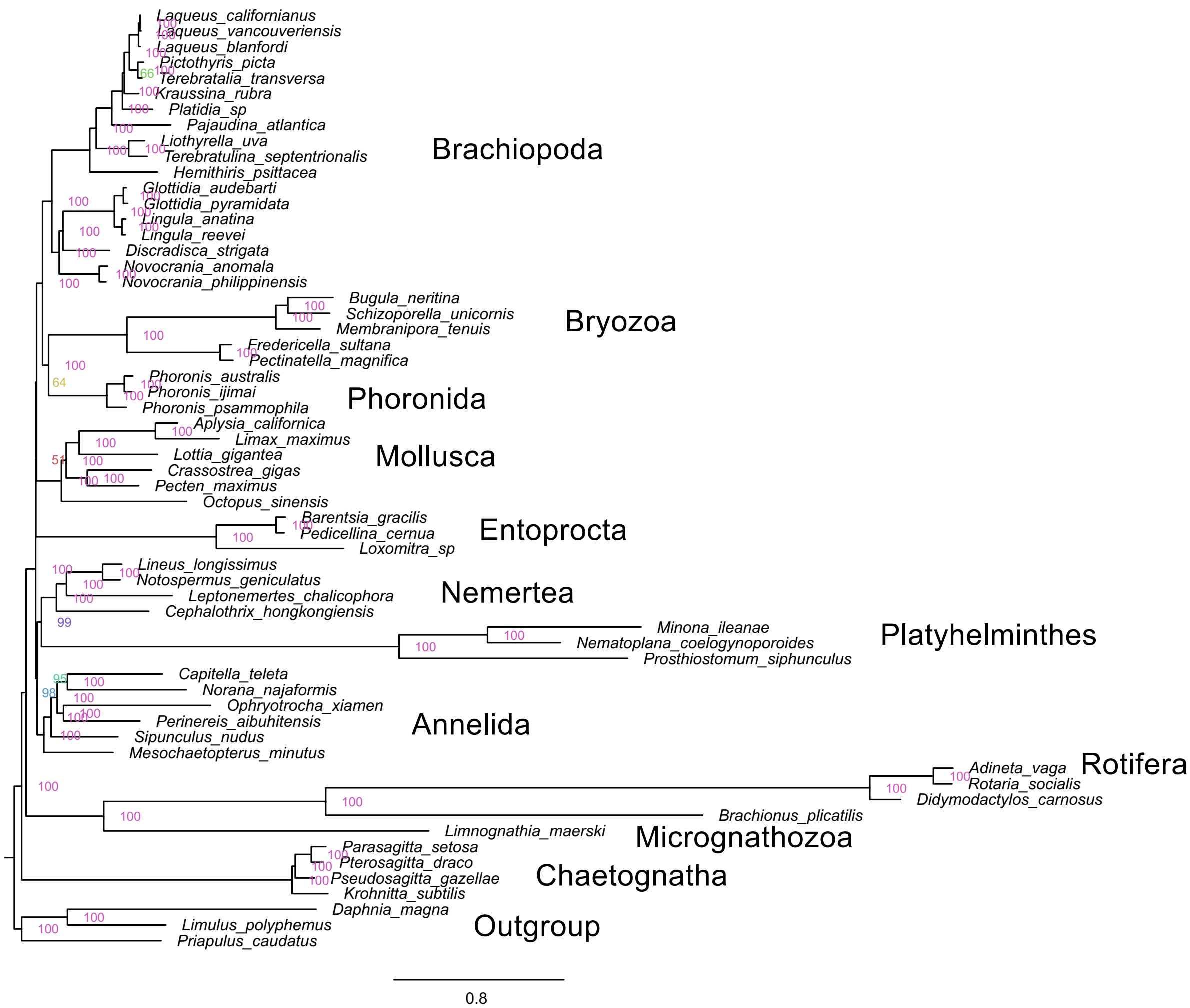

Fig. S5 Phylogenetic topology of Brachiopoda and Lophophorata within Lophotrochozoa under across-site compositional heterogeneous infinite CAT-PMSF model under (*31*) topology.

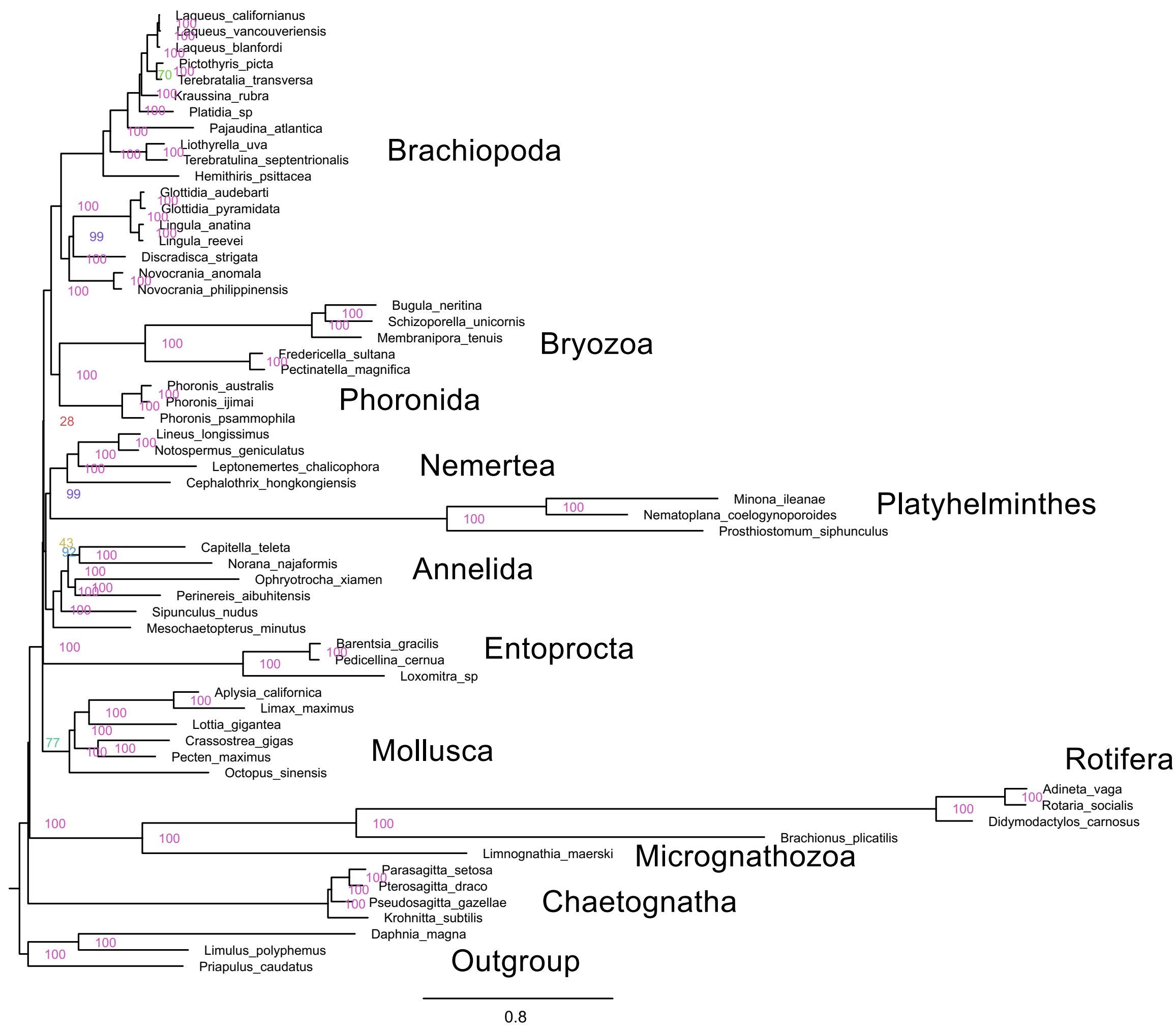

Fig. S6 Phylogenetic topology of Brachiopoda and Lophophorata within Lophotrochozoa under across-site compositional heterogeneous infinite CAT-PMSF model under (*30*) topology.

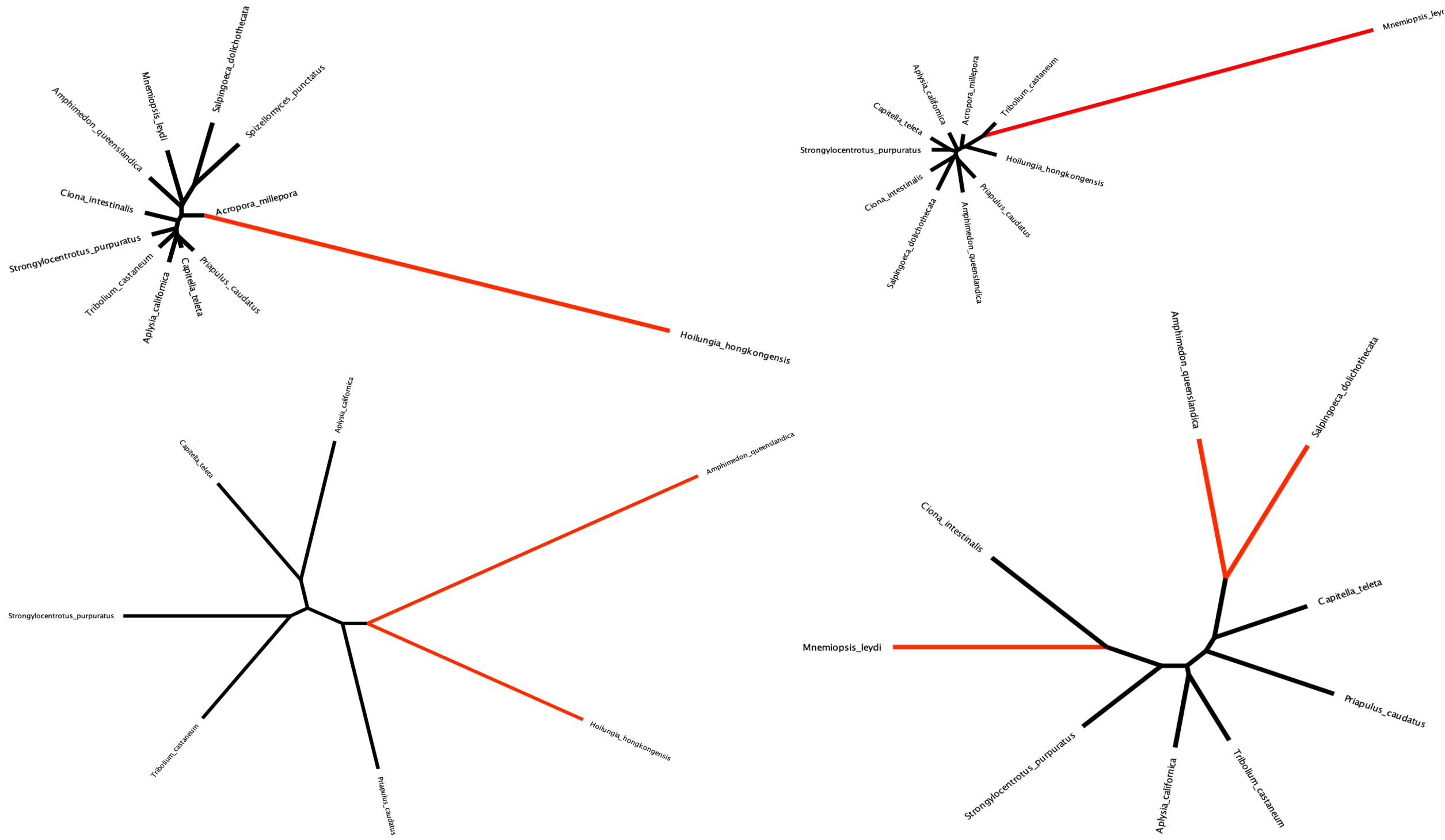

Fig. S7 Gene tree from the 672 sets where a sequence identified as paralogs has heterogeneous branch lengths.

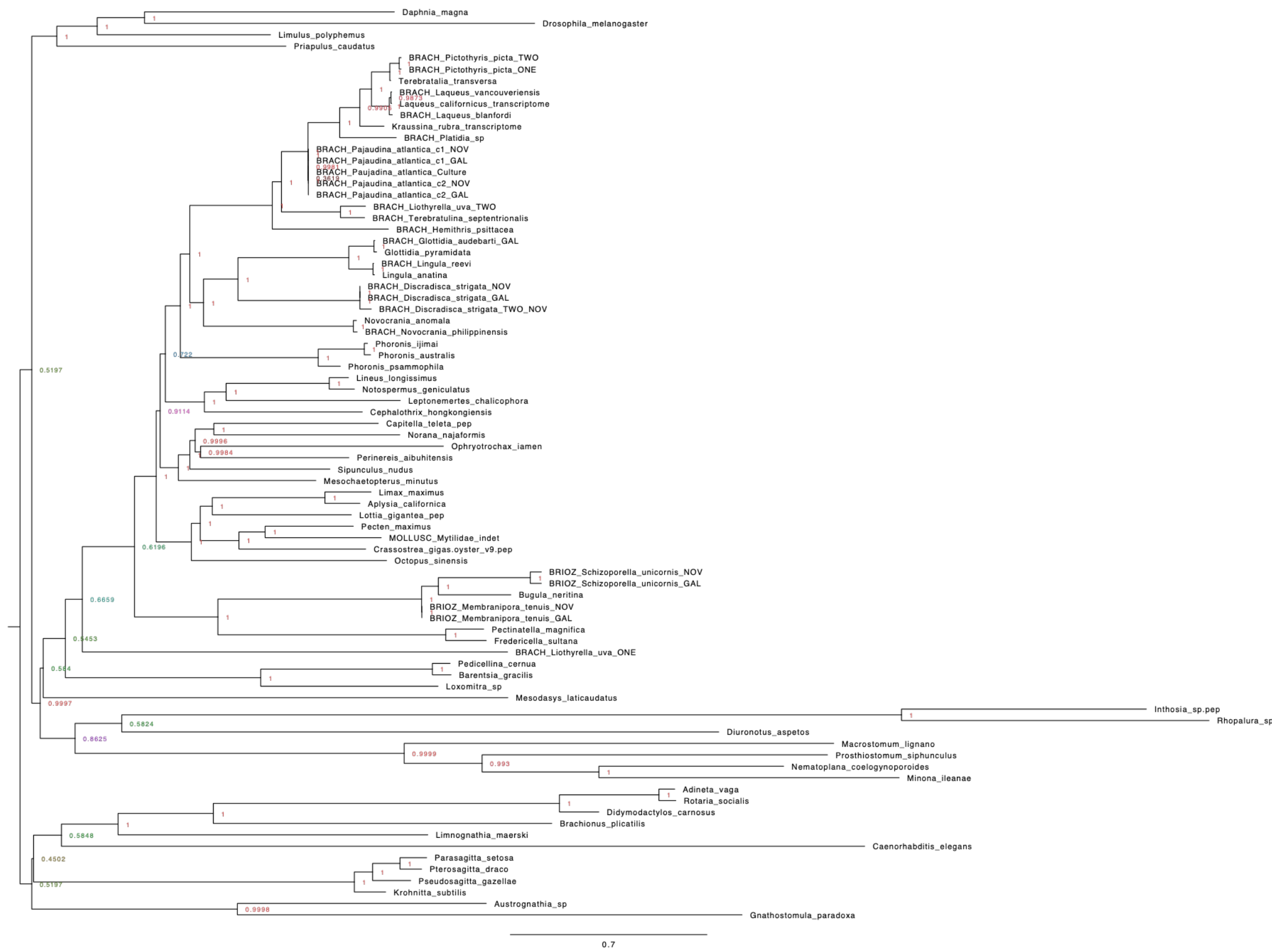

Fig. S8 Preliminary tree for data selection. We have sequenced 3 biological samples of *Pajaudina atlantica* across four sequencing batches that were run at different times. Two of these biological specimens were wild caught from Spain and one was lab cultured from GEOMAR facilities (Kiel). They were sequenced on different sequencing platforms as well: Runs 1-3 are on HiSeq and Run 4 is on NovaSeq platform. Our exploratory analyses show these four *Pajaudina atlantica* transcriptomes group together and sit at the same position (i.e., monophyletic and with negligible internal branch lengths), which we consider as strong evidence for no cross-contamination present. For the final analyses we selected only the cultured one as it has highest score of BUSCO completeness (i.e. for *Paujadina atlantica* with *Culture* suffix is the one we chose).

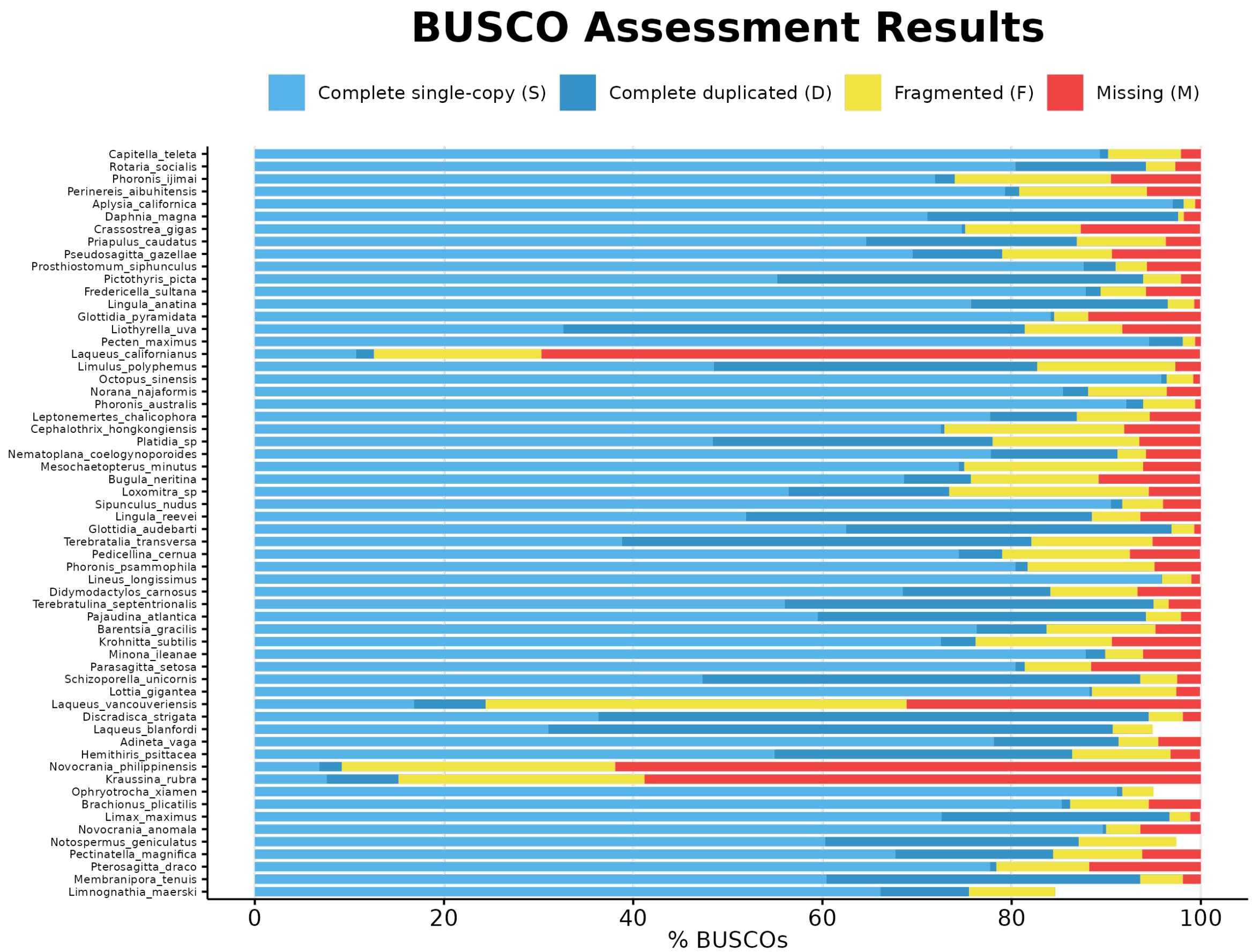

Fig. S9 BUSCO completeness for the dataset. With bars showing the percentage of Complete-single-copy, Complete-duplicated, Fragmented, and Missing genes.

Tables. S1-S6 (separate file)

Table S1- Sampling list of 60 proteomes, a list of all the taxa in the dataset and accession numbers
Table S2- BUSCO refining strategy reference genomes used
Table S3- Decontamination list, taxa and families
Table S4- Curated BUSCO families list, 301 families
Table S5- Collection information for new transcriptomic data
Table S6- Median information for 217 metazoa dataset
